# Single-cell ATAC-seq analysis of human embryoid bodies reveals crucial transcription factor networks involved in early germ layer specification

**DOI:** 10.1186/s13578-026-01561-8

**Published:** 2026-03-29

**Authors:** Linying Li, Xiaoyu He, Feng Zhang, Xiaodong Cai, Qiuhui Zhang, Tie Yang, Ran Tong, Shuang Li, Xiaoyan Ding, Yang Dong, Junmei Zhou, Lingjie Li

**Affiliations:** 1 Department of Central Laboratory, Shanghai Children’s Hospital, Shanghai Jiao Tong University School of Medicine, Shanghai, 200062 China; 2 Department of Histoembryology, Genetics and Developmental Biology, Shanghai Key Laboratory of Reproductive Medicine, Key Laboratory of Cell Differentiation and Apoptosis of Chinese Ministry of Education, Shanghai Jiao Tong University School of Medicine, Shanghai, 200025 China; 3 Center for Excellence in Molecular Cell Science, Chinese Academy of Sciences, Shanghai, 200031 China; 4 Digital Technologies, Hainan Bielefeld University of Applied Sciences, Hainan, China

**Keywords:** scATAC-seq, Human embryonic stem cells, Germ layer specification, Transcription factor network

## Abstract

**Background:**

Primary germ layer formation is a crucial event that follows fertilization and is accompanied by significant epigenetic changes. However, the precise mechanism of cell fate specification remains inadequately defined.

**Methods:**

In this study, we employed human embryoid body as an in vitro model to mimic early development and conducted single-cell ATAC sequencing to delineate lineage-specific chromatin regulatory elements and their associated transcription factor (TF) networks.

**Results:**

By assessing the consistency of TF gene activity and binding in each cell, we classified the three germ layers and performed pseudotime analysis, leading to the identification of “early committed cells” at the onset of each germ layer. Subsequently, we established TF regulatory networks by integrating the inferred loop signals (InferLoop) of binding site and target gene promoters, pinpointing the key TFs that govern the early committed cells in each germ layer. Finally, through gene knockdown and phenotypic analysis, we confirmed their essential roles in early cell fate determination.

**Conclusions:**

Our findings demonstrate that scATAC-seq can delineate transitional cell populations and identify a series of key TFs crucial in early germ layer determination. Furthermore, our methodological framework presents a pathway for future explorations in this intricate field of biology and provides valuable resources for mechanistic study. Collectively, this work provides novel insights to the intricate process of cell differentiation and germ layer specification, potentially contributing to significant advancements in the field of developmental biology research.

**Supplementary Information:**

The online version contains supplementary material available at 10.1186/s13578-026-01561-8.

## Background

Under natural differentiation conditions, stem cells frequently undergo various subtypes or exhibit distinct biological states, including the transition between quiescent and actively proliferating states as they develop into the organs with specific physiological functions. Furthermore, the multi-lineage differentiation processes always entail the involvement of distinctive intermediate cell subpopulations [1]. Nevertheless, the complexity and heterogeneity of individual cells pose significant challenges in the realm of developmental biology. Fortunately, advancements in single-cell isolation and barcoding technologies have facilitated the comprehensive detection and quantification of molecular variations at a single-cell level. These methodologies have yielded valuable insights into diverse biological processes, such as developmental plasticity [1, 2] and cancer heterogeneity [3–5].

Cell fate is determined through precise spatiotemporal control of gene expression patterns. Dynamic modification in *cis*-regulatory DNA elements, driven by changes in transcription factor (TF) binding, are instrumental in generating phenotypic diversity during development [6]. TFs directly decode genomic information by recognizing specific sequences and subsequently modulate gene expression, thus determining cell fate and developmental patterns [7, 8]. Acting as transcriptional regulators, lineage factors induce a sequence of genetic changes that ultimately lead to cell fate commitment during differentiation [9, 10]. Additionally, elaborate interconnected regulatory networks govern the patterns of gene expression regulation [11, 12]. Early explorations into cell fate regulation and lineage tracing have significantly enriched our understanding of the developmental processes. Nonetheless, a comprehensive understanding of cell identification at the transition time point and the TF regulatory network that orchestrates embryonic development remains elusive.

The human embryonic stem cell (hESC), firstly isolated from the inner cell mass (ICM), possesses the capacity for self-renewal, unlimited proliferation, and differentiation potential [13]. In addition to these attributes, their accessibility and ease of manipulation make hESCs a promising alternative for studying human developmental process [14]. Embryoid body (EB) is a three-dimensional structure derived from hESC through self-organization, containing three germ layers under suitable culture condition [15, 16]. By employing the EB experimental model to mimic early embryonic development, researchers have unveiled the signals and TFs that govern the differentiation events. Compared to directed differentiation protocol, a spontaneous EB model is informative for revealing lineage-specific transcriptional and epigenetic profiles, thereby providing deeper insights into embryonic development in vivo. EB is a highly heterogeneous structure comprising different germ layers, which has been analyzed at the single-cell transcriptome level to identify key TFs and signaling pathways directing the differentiation process; however, few studies have focused on the mechanism of transcriptional regulation [17, 18].

Cutting-edge single-cell assays, such as single-cell assay for transposase-accessible chromatin using sequencing (scATAC-seq), have unveiled valuable insights into chromatin states, representing active or repressive regulatory genomic regions, thereby shedding light on cell fate commitment [19, 20]. Ziffra et al. conducted scATAC-seq in primary human forebrain tissues and cerebral organoids, unraveling the developmental mechanisms in terms of chromatin states underlying the emergence of diverse neuronal lineages and cell fate specification, which further identified a converge of the accessible genomic loci during neuronal developmental with variants associated with neuropsychiatric disorders [21]. In addition to these capabilities, these advanced techniques have illuminated an array of interconnected epigenetic features including the specificity of TF binding events. Finkbeiner et al. integrated the scATAC-seq and transcriptomic analyses of the fetal human retina and successfully constructed the cell-type-specific regulatory networks and unveiled the sequential cascades of TFs that determine specific cell identities [22]. The advent of single-cell epigenome sequencing technologies has been particularly transformative in the field of stem cell biology. These methodologies have been instrumental in unraveling the complexity of epigenetic regulation at the individual cell level, offering unprecedented insights into the mechanisms of stem cell pluripotency, lineage commitment, and differentiation. Recently, we have developed an effective and precise method, InferLoop, to infer the strength of cell-type-specific chromatin connections using scATAC-seq data, which has proven to be helpful in building gene regulatory networks in the complex background of cell-type heterogeneity [23].

In this study, we performed scATAC-seq profiling of early differentiated cells in EBs at various time points, with an aim to delineate cellular heterogeneity, trace lineage-specific trajectories, and identify transitional cell states during the initial stages of three germ layer formation. We identified and characterized these early committed cells based on the consistency between the gene activity of specific TFs and their binding activity in individual cells. This hitherto unrecognized population of transitional intermediate progenitors within the early germ layer lineage emerges as a potential progenitor for the endoderm, mesoderm, and ectoderm layers, providing novel insights into cellular lineage commitment. To grasp the intricate regulatory network underlying the development of such population, we advanced our investigative framework by integrating inferred loop signals to construct an early-stage TF regulatory network. This approach enabled the identification of several novel TFs implicated in germ layer specification. To validate the functional relevance of these putative TFs within our predicted regulatory network, we conducted targeted perturbations through siRNA mediated gene knockdown. The resulting phenotypic alterations confirmed their functional roles, validating our predictions and underscoring their significance in early developmental processes.

## Materials and methods

### hESCs culture

hESCs (H9 cell line, from WiCell Research Institute, Madison, WI, https://www.wicell.org) were maintained in mTeSR1 (STEMCELL Technologies, Canada)/Matrigel (STEMCELL Technologies, Canada) and passaged using Accutase (STEMCELL Technologies, Canada) or 0.5 mM EDTA (STEMCELL Technologies, Canada).

### EB formation

hESCs were seeded in a feeder-free system using Matrigel hESC-Qualified Matrix (STEMCELL Technologies, Canada) and were maintained in mTeSR1 (STEMCELL Technologies, Canada). EBs were spontaneously differentiated in E6 medium (STEMCELL Technologies, Canada) [24]. Briefly, to induce the formation of EBs, hESCs were dissociated into single cells and then seeded on Matrigel-coated 6-well plate, where they were cultured in mTeSR1 for 4 days. After 4 days of culture, hESCs were digested using Accutase for 5–7 min, and then about 3 × 10^6^ cells were loaded to a single well of AggreWell^TM^800 plate (STEMCELL Technologies, Canada) pretreated with Anti-Adherence Rinsing Solution (STEMCELL Technologies, Canada), resulting in 10,000 cells/microwell. The AggreWell^TM^800 plate was then centrifuged to capture the cells in the microwells, and the cells were incubated at 37 °C for 24 h. The day after seeding, EBs were harvested from the AggreWell^TM^800 plates and transferred to an ultra-low attachment six-well plate (Corning, America). Daily partial (3/4)-medium changes were performed until EBs were harvested.

### Three germ layer differentiation assays

Mesendoderm (ME) differentiation and the neural ectoderm differentiation of the hESC were performed based on the published protocol described by Xie et al. [25] and Fang et al. [26], respectively. H9 hESCs were plated on to Matrigel-coated plates and maintained in mTeSR1 media. To induce differentiation into mesendodermal cells, the cells were fed with “mesendoderm differentiation medium” containing 5 ng/mL BMP4 (PeproTech, America) and 25 ng/mL Activin A (PeproTech, America) in Essential 8 media (Gibco, America) for three days, thereby forming mesendodermal cells. To induce differentiation into neural ectodermal cells, the cells were fed with “neural induction medium” containing 1:100 N2 (Gibco, America), 1:50 B27 without Vitamin A supplement (Gibco, America), 1:100 Nonessential amino acid (Gibco, America), 1:100 Penicillin/Streptomycin (Gibco, America), 5 μM SB431542 (Selleck, America), 50 nM LDN193189 (Selleck, America) in 1:1 NeuroBasal medium (Gibco, America): DMEM/F12 (Gibco, America) for four days, thereby forming neural ectodermal cells.

### Small interfering RNA (siRNA)-mediated gene knockdown in ESCs

To knock down the expression levels of TCF12, EBF1 and NFIC, H9 hESCs were plated on 6-well plates and were transfected with 100 nmol of siRNAs (Biosune, China) using Lipofectamine RNAiMAX Reagent (Invitrogen, America) with a standard protocol. The siRNA oligonucleotides were designed, synthesized and fluorescence-labelled by Biosune. The transfection experiment was performed over three times, and the knockdown efficiency was measured by real-time quantitative PCR (RT-qPCR). In each batch of the experiment, there are three target gene siRNA and one control siRNA oligos transfected in three replicate wells. All siRNA including negative control (NT) siRNA sequences are listed in Table S1.

### scATAC-seq library preparation and sequencing

For each scATAC-seq sample, Day 4 (D4) EB and Day 8 (D8) EB were digested in Accutase (STEMCELL Technologies, Canada) at 37 °C for 20 min and gently pipetted no more than twice. Dissociation was stopped by adding DMEM/F12 (Gibco, America), and the cell suspension was centrifuged at 300 × g for 5 min. The cells were then washed and resuspended in D-PBS (Gibco, America) twice and centrifuged at 300 × g for 5 min. The cell pellet was resuspended in D-PBS and counted using a Countess II automated cell counter (Invitrogen, America).

scATAC-seq libraries were generated according to the protocol modified from Buenrostro et al. [27] and Xu et al. [28]. We recommended starting with 100 k cells. Firstly, the cells were fixed with formaldehyde (Invitrogen, America) at a final concentration of 0.1%. The fixation was stopped through washing the fixed cells twice with 0.5 mL PBS. The omniATAC-seq was then performed, and transposed nuclei were resuspended in nucleus dilution buffer and kept on ice until sorting. A 96-well plate was prepared for sorting by adding Reverse Crosslink + Proteinase K (Invitrogen, America) to each well. Fluorescence activated cell sorting (FACS) was used to dispense one cell in each well for all 96 wells. The plate was incubated in a thermal cycler at 55 °C for 4–16 h, followed by incubation at 85 °C for 5 min to inactivate Proteinase K. The reaction was quenched by adding 10% Tween 20. PCR Mix is then prepared, and each well contains 10 μL 2 × NEB Master Mix (New England Biolabs, America), 1 μL Ad1.X Primer, 1 μL Ad2.X Primer. The cycling conditions were 72 °C for 5 min, 98 °C for 30 s, followed by 27 cycles of [98 °C for 10 s, 63 °C for 30 s, 72 °C for 1 min], with a final hold at 4 °C. Note that each sample plate should be marked with a single Ad1.X barcode across all 96 wells and each well of a plate should be marked with a unique Ad2.X barcode. When all plates have been completed, the ATAC products in each well were pooled and purified using DNA Clean & Concentrator™-5 (ZYMO, America). There are some modifications in this protocol. First, the PCR round was reduced to one, which was used to add adapters and amplify, efficiently avoiding the PCR bias. Second, the unique cell identity designed for each cell was labelled by two barcodes, one is called i5 and the other called i7. Third, only common reagents and equipment were required, which reduced the cost, increased the prevalence and reduced the time spent. Fourthly, in the absence of exonuclease I, we reduced the small fragment residue by adjusting the adapter concentration. Quality control of the sequencing libraries was performed on the Agilent Bioanalyzer 2100, and library yield was quantified using the Qubit DNA high sensitivity assay (Invitrogen, America). Sequencing was performed on an Illumina Novaseq PE150 system operated by the Shanghai Personal Biotechnology Cp. Ltd. The primers used for the scATAC-seq library are listed in Table S2.

### scATAC-seq analysis pipeline

Sequencing data was used as an input to the standard single-cell Analysis of Regulatory Chromatin in R (ArchR; https://github.com/GreenleafLab/ArchR) analysis pipeline [29]. Briefly, the cells with a mapped ATAC-seq fragment number between 10^4.5^ and 10^5^ were retained for creating Arrow files. Doublets were then inferred. After creating ArchR Project, the cells with log_10_Reads in TSS between 3.5 and 4.5 were retained, and doublets were removed, resulting in 474 cells. Iterative Latent Semantic Indexing (LSI) dimensionality reduction was performed through the *addIterativeLSI* function, and Uniform Manifold Approximation and Projection (UMAP) was used to visualize the attributes of cells. The ChromVAR R package was used to provide motif annotations and measure TF motif enrichments (binding activity matrix of TF) at the single-cell level in scATAC-seq data [30]. The gene activity matrix was generated using ArchR. Gene activity was used to estimate the expression levels of specific genes based on the accessibility of regulatory elements located in close proximity to the gene of interest. Consistency scores were introduced to reflect the contributions of TFs in individual cells, which were generated using the ‘*inferloop.calILS*’ function implemented in InferLoop [23] with the gene activity matrix and the binding activity matrix of TFs as inputs. The calculation of consistency scores is based on the InferLoop framework, which constructs a metric to quantify inferred loop signals from a matrix of peak signals and a list of predicted loops. The regulatory network was constructed based on the loop signals (InferLoop [23]) and consistency score of TFs. The regulatory networks were visualized in Cytoscape [31]. Note, for the construction of regulatory network, weight of each edge was calculated based on loop signals (between TF motif sites and target’s transcription start sites) and consistency score of TFs.

### Immunofluorescence analysis

hESCs and differentiated cells were grown on tissue culture-treated coverslips in 4-well plates (ThermoFisher Scientific, America) and fixed with 4% paraformaldehyde for 15 min at room temperature. The cells were then washed three times with PBS. The cells were penetrated with 0.2% TritonX-100 (Sangon, China) for 30 min and then blocked with 10% donkey serum (Solarbio, China) for 60 min at room temperature. Next, cells were incubated with primary antibodies in a humidity chamber at 4 °C overnight, washed three times with PBS and then incubated with fluorophore-conjugated secondary antibodies for 2 h in the dark at room temperature. The cells were then washed with PBS and stained with DAPI (Beyotime, China). Imaging was performed using a LEICA TCS SP8 X motorized inverted microscope. Primary antibodies and their dilutions were used as follows: anti-OCT4 (Abcam ab181557, 1:250); anti-Nanog (SantaCruz sc-293121, 1:200); anti-Brachyury / Bry / T (Abcam ab209665, 1:1000); anti-PAX6 (Abcam ab195045, 1:350); anti-SOX1 (Abcam ab109290, 1:100); anti-TBR2 / Eomes (Abcam ab216870, 1:100); anti-GATA6 (CST 5851, 1:1600); anti-SOX17 (Abcam ab224637,1:500). Secondary antibodies conjugated to either Alexa Fluor 488 or 555 fluorophores (ThermoFisher Scientific, America) were used at a 1:500 dilution. Each immunofluorescence staining experiment was performed with 3 independent biological replicates.

### RT-qPCR

Total RNA was purified from samples using the standard TRIzol method. Complementary DNA (cDNA) was prepared using HiScript® III RT SuperMix (Vazyme, China) with 1 μg of total RNA. Reverse transcription was performed using standard cycling conditions according to the manufacturer’s instructions. cDNA was diluted tenfold, and qPCR was performed using ChamQ Universal SYBR qPCR Master Mix (Vazyme, China). The relative gene expression was calculated using the ^△△^Ct method and plotted based on the relative gene expression. The primers used for qPCR are listed in Table S3. Each RT-qPCR experiment was performed with 3 independent biological replicates, and each assay included 3 technical replicates.

### Flow cytometry analysis of EBs

After 4 days of culture, EBs were harvested for analysis. The EBs were carefully aspirated to remove the culture medium and washed once with PBS. Then, 1 mL Accutase was added, and the EBs were digested for 5 min in a 37 °C incubator. The digestion was stopped by adding DMEM medium supplemented with 10% fetal bovine serum. The resulting cell suspension was centrifuged at 200 g for 5 min, and the supernatant was discarded. Subsequently, the cells were resuspended in 2.5 mL pre-cooled PBS, and a total of 7.5 mL pre-cooled absolute ethanol was added slowly and incrementally with gentle mixing during each addition. The cell suspension was then fixed at 4 °C for 30 min.

After fixation, the samples were centrifuged at 200 g at room temperature for 5 min to discard the ethanol fixative. The cells were resuspended in 5 mL PBS and centrifuged again to complete one wash. Following removal of the supernatant, a permeabilization buffer of 0.1% PBST (containing Triton X-100) was added, and permeabilization was performed at room temperature for 30 min in the dark. After permeabilization, the samples were centrifuged at 400 g for 5 min, the supernatant was discarded, and the cells were washed by resuspending in 5 mL PBS followed by centrifugation. This wash step was repeated twice. Next, primary antibodies were diluted in PBS according to the manufacturer’s instructions. The cells were incubated with the primary antibodies at room temperature in the dark for 1 h. After incubation, the samples were centrifuged at 400 g for 5 min, the supernatant was discarded, and unbound primary antibodies were removed by washing twice with PBS through resuspension and centrifugation. Subsequently, fluorescently labelled secondary antibodies were diluted in PBS and incubated with the cells at room temperature in the dark for 1 h. After incubation, the samples were centrifuged at 400 g for 5 min. The supernatant was discarded, and the cells were washed twice with PBS through resuspension and centrifugation. Finally, the stained samples were subsequently analysed by flow cytometry using a Beckman Coulter CytoFlex S Flow Cytometer. Primary antibodies and their dilutions were used as follows: anti-SOX17 (CST 81778S, 1:3000); anti-GATA6 (R&D AF1700, 1:200); anti-EOMES (Abcam ab216870, 1:500) and anti-PAX6 (Selleck A5371, 1:200). Secondary antibodies conjugated to either Alexa Fluor 488 or 555 fluorophores (ThermoFisher Scientific, America) were used at a 1:1000 dilution. Three independent biological replicates were performed for each TF knockdown assessment.

### Statistical analysis

Data are presented as mean ± SEM. Statistical significance of differences between two groups was assessed using either an unpaired two-tailed Student’s t-test or a paired t-test, as indicated in the respective figure legends. Statistical significance was defined as *p < 0.05, **p < 0.01 and ***p < 0.001. *p* < 0.05 was considered statistically significant. The number of independent experiments (biological replicates) for each experiment is provided in the respective figure legends.

## Results

### The high quality scATAC-seq data reveals the heterogeneity during early differentiation of human embryonic stem cells (hESCs)

**Fig. 1:**
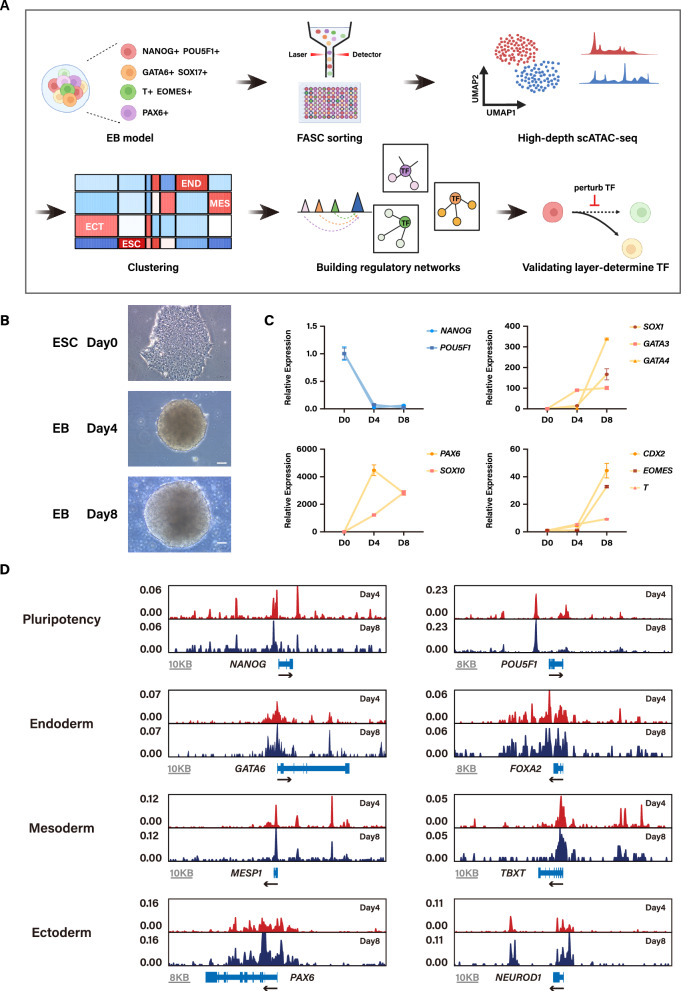
Overview of scATAC-seq analysis on hESC early differentiation. **A** Schematic diagram summarizing the overall experimental flow integrating iterative bioinformatics and experimental validation. Detailed information about this study can be found in the Results and Methods sections. **B** Morphological changes of differentiating EBs at days 4 and 8 in E6 medium. Scale bar = 100 μm. **C** Expression level in replicates of several pluripotency and early differentiation markers for ESC and EB measured by RT-qPCR. RT-qPCR experiments were performed with 3 independent biological replicates. Data are presented as mean ± SEM. **D** Comparison of peak signal obtained from Day 4 and Day 8 EBs at a locus containing the TSS of pluripotent and three germ-layer specific markers. y-axis: Norm. ATAC signal range

To investigate the dynamics of chromatin accessibility during the early stages of human development, we initially employed a simple yet effective approach to cultivate EBs from hESCs, maintaining them in E6 medium to allow for spontaneous differentiation. The differentiation status of the EBs was subsequently confirmed through their morphological characteristics, coupled with RT-qPCR analysis of specific lineage markers (Fig. 1B, C). As shown in Fig. 1C, a decrease in the expression of pluripotent markers and a notable increase in differentiation markers were observed at two time points (D4, D8), as compared to the undifferentiated stage (D0). This observation indicates that EB model can recapitulate early human three germ layer differentiation.

In order to comprehensively capture the epigenetic heterogeneity during hESC early differentiation, EBs were prepared as single-cell suspension, and we conducted plate-based and FACS-based scATAC-seq to elucidate cell-lineages specific chromatin dynamics (Fig. 1A). This protocol was modified from a previously reported plate-based single-cell ATAC protocol [28]. In brief, the EBs were dissociated into a single cell suspension, followed by cell lysis and permeabilization of the nuclei. Subsequently, a hyperactive Tn5 transposase was employed to integrate sequence adaptors into accessible chromatin regions devoid of nucleosome occupancy. These steps were conducted at a bulk level. The transposed cell suspension was then individually isolated into each well of 96-well plates containing sodium dodecyl sulfate (SDS) using FACS. Upon denaturation and release of the Tn5 transposase, fragment insertion occurred. Tween 20 was introduced to counteract the negative impact of SDS on subsequent PCR reaction. Both plate and well barcodes were added to each well in the same round of PCR. Finally, samples from different plate were pooled together and purified for sequencing.

The scATAC-seq library was sequenced and the resultant dataset was analyzed using the computational package ArchR [29]. First, the datasets were filtered to retain high-quality nuclei by setting thresholds based on the number of unique nuclear fragments, and the transcription start site (TSS) enrichment score (Supplementary Fig. 1C). This process aimed to enrich cells exhibiting a higher fraction of fragments mapping to the TSS as compared to other genomic locations. The sequenced data demonstrated a high read depth, with a median of approximately 100 k fragments per cell. The TSS enrichment score reached nine, confirming the outstanding performance of our improved scATAC-seq protocol (Supplementary Fig. 1C). Subsequently, the datasets underwent doublet discrimination, resulting in a final dataset comprising a combined total of 472 high-quality nuclei across the two samples (295 single cells from D4 EBs and 177 single cells from D8 EBs) (Supplementary Fig. 1B). Furthermore, we observed that the track at locus of marker genes associated with germ layer differentiation was generally more accessible in the respective cell populations (Fig. 1D, Supplementary Fig. 1A). This observation underscores that the excellence signal-to-noise ratio achieved by our scATAC-seq library, and supports the assertion that our model accurately captured the differentiated state.

### Joint assessment of the accessibility level and binding activity unveils lineage-specific TFs

**Fig. 2:**
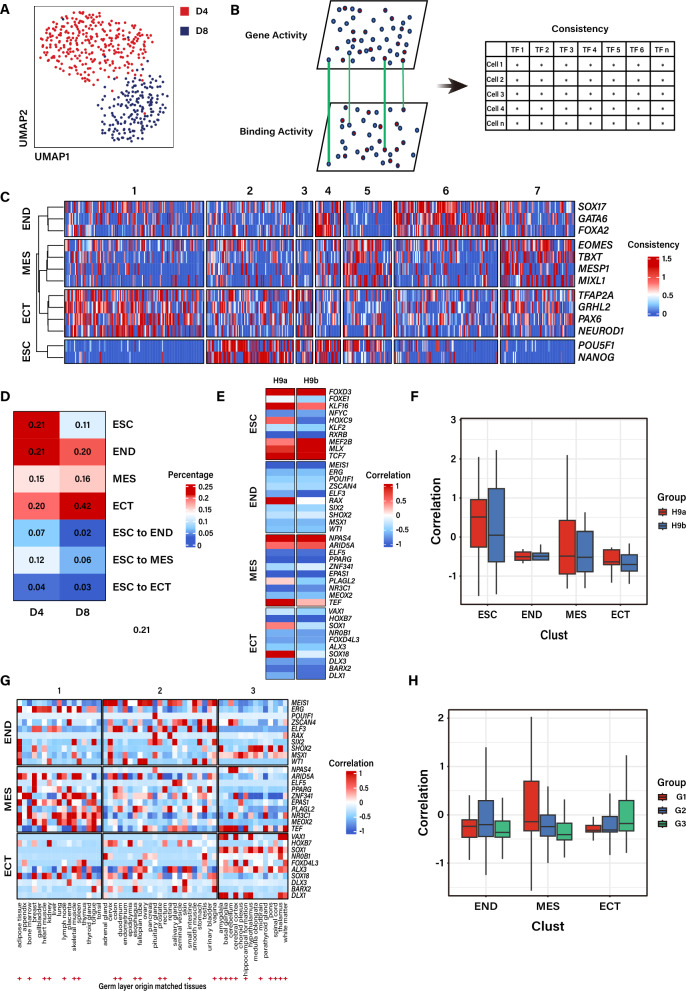
Joint assessment of the consistency of accessibility level and binding activity reveals lineage-specific TFs. **A** UMAP visualization of the scATAC-seq data, illustrating the separation between D4 and D8. **B** Diagrams illustrating the principle of clustering strategy. **C** Heatmap displaying the different clusters identified using consistency scores. The color scale represents the consistency scores of TFs in each cluster. **D** Heatmap showing the proportion of cells in each cluster within each stage. The color scale represents the cell proportion. **E**–**H** Heatmap and boxplots showing the normalized expression levels of novel TF using z-score in the published RNA-seq data from H9 hESCs (**E**–**F**) and adult tissues (**G**–**H**). ESC, embryonic stem cell; END, endoderm; MES, mesoderm; ECT, ectoderm

The dataset was then subjected to dimensional reduction, clustering, and visualization in a 2D UMAP embedding. Initially, we attempted to cluster cells using unsupervised clustering methods but failed to separate them into the expected three populations representing endoderm, mesoderm and ectoderm as expected. The UMAP projection showed that cells from different time points exhibited distinct distribution (Fig. 2A), indicating that these cells at different stage possess diverse genomic features corresponding to their developmental stage.

An inevitable limitation of the information derived from scATAC-seq data is not fully reflecting the actual gene expression pattern, presenting a primary challenge in scATAC sequencing data. To address this issue, a common strategy in regular analysis is to integrate the scRNA-seq data from the same biological system to aid in interpreting the scATAC-seq data [29, 32]. Nevertheless, not all studies possess the requisite matching expression patterns, and the reliable methods for clustering cell populations and annotating cell type based solely on scATAC-seq information remain unexplored. In this study, in our pursuit to obtain a robust peak signal to build an atlas of chromatin open region related to cell-type specific lineage, thereby disclosing the genomic dynamics and key regulators of hESC differentiation, we integrated the open degree of TSS represented by the TSS signal and TF binding activity reflected in the TF motif enrichment analysis by ChromVAR [30] (Fig. 2B). The combined evaluation of the open degree of TSS and the motif activity collectively determines the final score, defined as the consistency of TF (Fig. 2B, Supplementary Fig. 2A, B, C), all of which are derived from the scATAC-seq data.

Subsequent analysis was conducted based on the consistency of each TF. Having obtained the consistency score of each TF, we proceeded to cluster and annotate cell populations using selected well-known TFs corresponding to a specific cell type during early differentiation, based on the consistency (accessibility of the gene promoter plus the binding activity) of the following marker genes: *SOX17*, *GATA6* and *FOXA2* for endodermal cells (END); *EOMES*, *TBXT*, *MESP1* and *MIXL1* for mesodermal cells (MES); *TFAP2A*, *GRHL2*, *PAX6* and *NEUROD1* for ectodermal cells (ECT); *POU5F1* and *NANOG* for embryonic stem cell (ESC). We then employed unsupervised clustering method K-means to retain as much realistic data information as possible. Considering that there might be an intermediate stage differentiating from ESC to three lineage cell types, we set k = 7 to force all cells to be automatically clustered into 7 cell populations (Fig. 2C). In addition to the four well-defined populations (ESC, END, MES, ECT), we observed three undefined populations characterized by the simultaneous display of typical markers of both ESC and one germ layer in terms of the chromatin state, hence named as “the early committed cells”. Meanwhile, we compared the percentage of different cluster at two differentiation stages (Fig. 2D). Consistent with normal developmental dynamics from D4 to D8, there was an increase in the proportion of differentiated cells, especially ectoderm, which reached up to forty-two percent. Undefined cells were abundant at D4 (Fig. 2D), and clustering indicated that these undefined populations were enriched in the consistency score of well-known marker TFs, characterized by both a pluripotent state and a differentiated state (Fig. 2C), suggesting that they were in a transitional phase. The signature of transition cells partially overlapped with that of both the pluripotent state and the differentiated state.

Moreover, we discovered novel TFs unique to three germ layers after clustering (Supplementary Fig. 3A, B, C). Based on the assumption of a significant correlation between gene expression level (RNA-seq data) and chromatin accessibility (ATAC-seq data), we sought to confirm the expression pattern of these novel TFs during cell differentiation. We examined the expression of these novel TFs, which could be reliably used to identify specific cell type population. We compared the expression pattern of defined cell populations in previous studies (Fig. 2E–H). Compared with published RNA-seq datasets from ESC [33], we found that certain TFs, such as FOXD3, KLF16 and TCF7, which demonstrated higher consistency score, exhibited similar high expression levels in the datasets (Fig. 2E, F). Furthermore, we clustered the RNA-seq data of various tissues stored in the Human Protein Atlas (https://www.proteinatlas.org) using novel TFs specific to three germ layers, and automatically clustered them into three populations (Fig. 2G, H). Within these populations, mesoderm-derived tissues such as adipose tissue and bone marrow were classified into the same cluster, and similar results could be observed for endoderm- and ectoderm-derived tissues.

These findings indicated that the novel TFs aligned with the expression pattern of the corresponding germ layers, thereby validating our clustering methods and suggesting that these TFs may potentially play a role in early lineage commitment. Altogether, our results demonstrated that our strategy, which combined TSS signal and TF binding activity, can yield highly reliable clustering outcomes.

### Identification of master TFs driving lineage commitment through the TF-TF transcriptional regulatory network

**Fig. 3:**
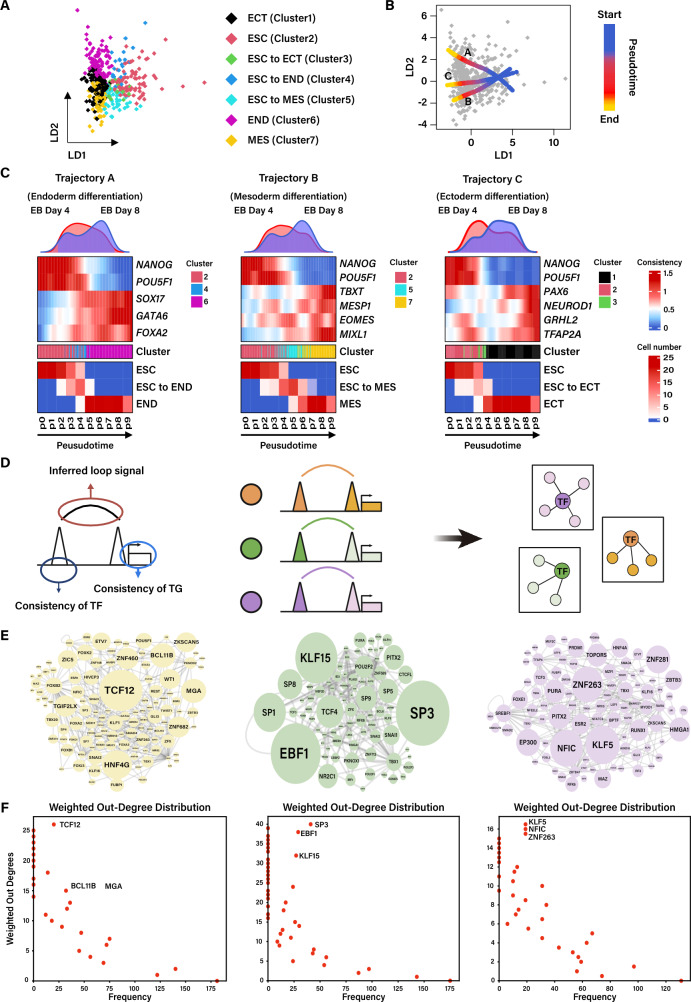
Transcriptional regulatory network of TFs during the transition phase of early differentiation progress identifies master TFs driving lineage differentiation. **A**, **B** Visualization of Linear Discriminant Analysis (LDA) using 7 clusters identified using the consistency scores of marker TFs. The cells are annotated by colors representing different clusters (**A**) or pseudotime orders (**B**). **C** Heatmap showing the characterizations of different lineage differentiation. Top: Cell density along the developmental trajectory. Middle: the consistency score of lineage-specific TFs during the developmental process. Bottom: Cell number along the developmental trajectory. **D** Diagram depicting the basic principle governing the establishment of the regulatory network. **E** Lineage specific TF-TF regulatory network. Left: ESC to END early committed cell population. Middle: ESC to MES early committed cell population. Right: ESC to ECT early committed cell population. **F** Weighted out-degree distribution of gene regulatory network referenced in (**E**). Left: ESC to END early committed cell population. Middle: ESC to MES early committed cell population. Right: ESC to ECT early committed cell population

Spontaneously differentiated EBs display heterogeneous patterns of differentiated cell types (Fig. 1C, D, Fig. 2C, Supplementary Fig. 1A), providing a diverse sample for pseudotime analysis. To unveil the single-cell genomic dynamics of each germ layer cell, we arranged single cells through EB differentiation and constructed a comprehensive lineage differentiation trajectory with a tree-like structure using the Linear Discriminant Analysis (LDA) algorithm for supervised dimensional reduction (Fig. 3A, B, Supplementary Fig. 4). The trajectory analysis predicted the ESC population as the starting point and depicted a trajectory through a transitional phase to the three main mature lineage branches: END, MES and ECT cells. Subsequently, we focused on the lineage-specific differentiation pseudotime trajectory and assessed the consistency score of germ layer-specific TFs in each group (Fig. 3C). As anticipated, some intermediate cells were indeed positioned in the middle of the trajectory from ESC to END, MES and ECT. Moreover, a higher percentage of differentiated cells were identified at later stage and they generally showed consistency with marker TFs. Beyond that, we observed that the pluripotent marker *POU5F1* disappeared later than *NANOG*, aligning with the previous studies [34, 35], and *EOMES* emerged earlier than other mesoderm marker. To elucidate the involvement of these TFs in transition phase, we integrated the developmental trajectory and depicted these cells according to their location of pseudotime analysis and divided them into ten bins due to the limited cell number. As indicated in the Fig. 3B, ESC cells were predominantly distributed at the early stage, with the consistency score of pluripotent TF markers, such as *NANOG* and *POU5F1*, showing higher level. In contrast, the differentiated cell population was positioned at the late stage with higher consistency scores of the respective TFs (Fig. 3C). As anticipated, we identified one bin in the middle of ESC and END, MES or ECT.

Prior studies have indicated that chromatin undergoes significant changes during early development, particularly in the distal regulatory regions [36–38]. Recognizing that numerous accessible regions are not typically found at promoters, we integrated loop information and utilized the InferLoop algorithm [23], developed by our laboratory, to posit the presence of a loop linking the distal peak containing TF binding site to the TSS of target genes (TG). In this context, our focus was directed solely on the downstream TF genes (Fig. 3A, B, C).

To construct a comprehensive TF network governing early lineage differentiation, we devised an algorithm that links changes in consistency score of TFs, the predicted loop signal between TF and TG, and the consistency score of TGs (Fig. 3D). The assumed loop signal was calculated based on Inferloop, a method for inferring the strength of chromatin interaction from scATAC-seq data [23]. Considering that the function of TF depends on the chromatin accessibility of its TSS, the motif binding activity and loop signal from distal regulatory elements, we created a TF-loop-TF model to integrate all genomic features obtained from scATAC-seq to the fullest extent. We then individually ranked all TF-loop-TF information and devised regulatory networks specific to the ESC stage and three germ layers. As shown in Supplementary Fig. 3D, many known TFs such as FOXA2 and SOX17 in END were accurately predicted. Other less-known factors like ras-responsive element binding protein 1 (RREB1), Zinc finger protein 460 (ZNF460) and Krüppel-like factor 7 (KLF7), which displayed high network degree, have also been reported in other studies [39–41]. For example, RREB1 had been found to participate in definitive endoderm (DE)-derived primitive gut tube differentiation from hESCs and be specifically expressed at this stage, as the expression of RREB1 in the primitive gut tube displayed higher levels than that in the mesodermal and neuroectodermal differentiation models [39]. However, there has been a lack of detailed research exploring the precise mechanisms of these TFs or clarified their functions during early development, especially during the stage of three germ layer formation. Following validation of the practicability and soundness of using TF regulatory network as a reference for identifying TFs that play a crucial role in a biological process, we proceeded to select TFs in these transitional cell population. Subsequently, we established a TF-TF regulatory network based on the aforementioned principle (Fig. 3E, Supplementary Fig. 5) and demonstrated the centrality of this network in identifying pivotal nodes (Fig. 3F).

### Disruption of the predicted TFs specific to the transition phase mitigates the early germ layer specification

**Fig. 4:**
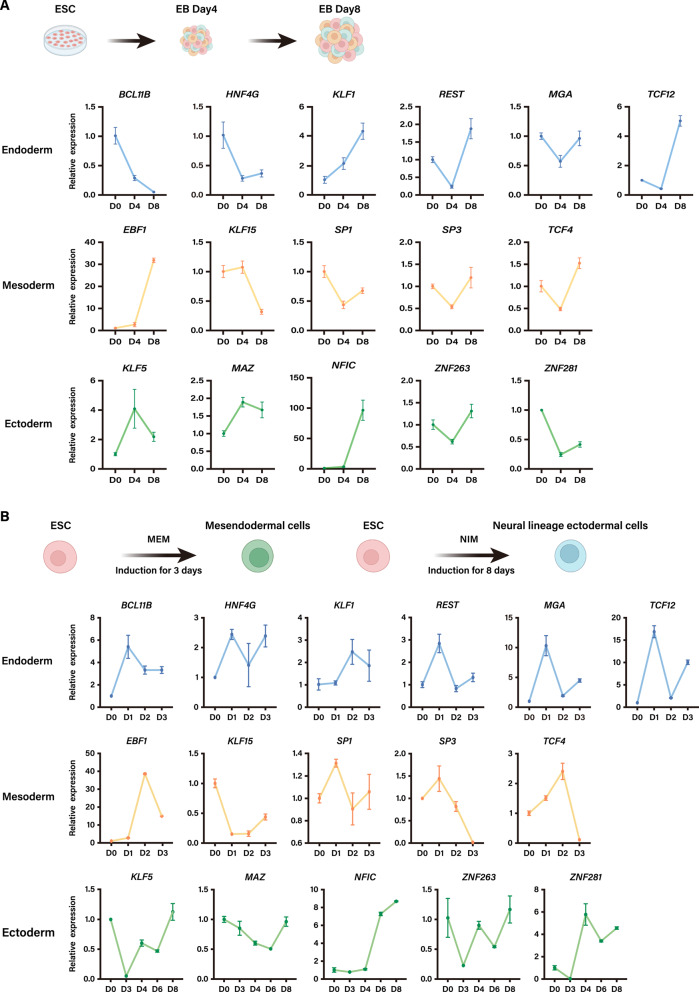
The expression patterns of the predicted regulatory TFs in EB model and directed differentiation culture system. **A** Temporal expression profiles of selected novel TFs in EB model as determined by RT-qPCR. **B** Temporal expression profiles of selected novel TF in the directed differentiation culture system as determined by RT-qPCR. RT-qPCR experiments were performed with 3 independent biological replicates. Data are presented as mean ± SEM

In our functional study, we successfully developed differentiation protocols for hESC line H9 into endoderm, mesoderm and neural progenitor cells, which had been validated by the expression of germ-layer-specific marker genes using RT-qPCR and immunofluorescence (IF) (Supplementary Fig. 6). Subsequently, we examined the mRNA expression of candidate TFs in the EB model (Fig. 4A) and the directed differentiation model (Fig. 4B). While some candidate TFs exhibited a consistent expression pattern with the marker TFs, most of them displayed low expression levels in the EB models, and a similar expression dynamic could not be seen in the EB model and directed differentiation condition. The discrepancy could be attributed to the following reasons. First, our RT-qPCR was performed in the bulk EBs, whereas these candidate TFs may be present in the cells of the transition phase, which constitutes only a small percentage of EBs. Second, the predicted candidate TFs were based on our EB model, which was cultured E6 medium, while the RT-qPCR validation was performed in directed differentiation condition.

**Fig. 5:**
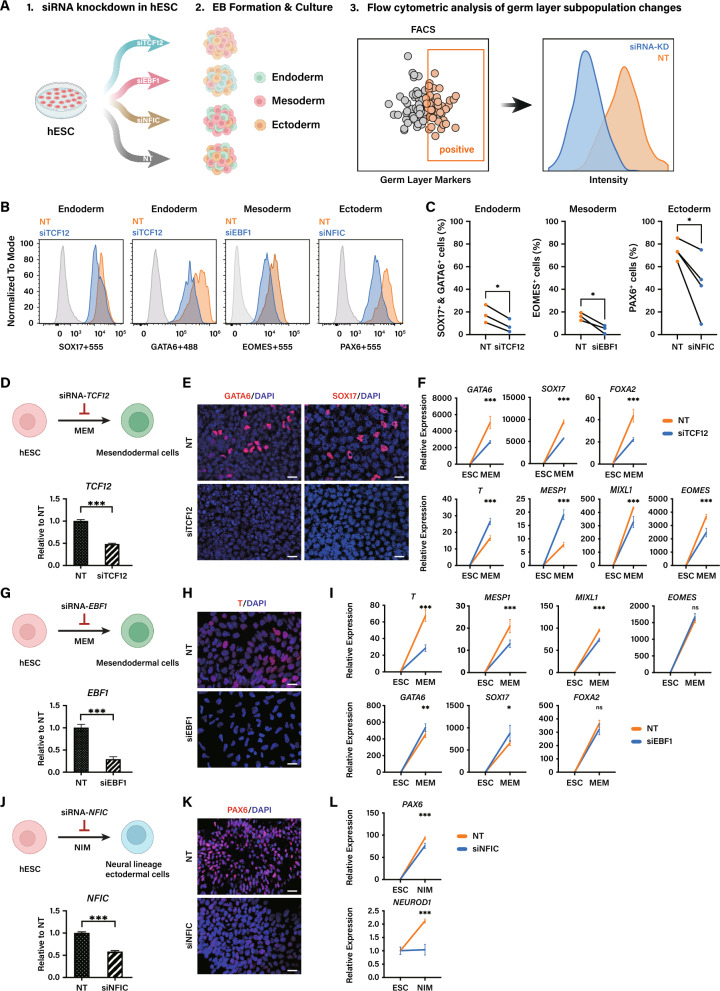
Perturbation of *TCF12*, *EBF1* and *NFIC* disrupts germ-layer specification in both EB and directed differentiation systems. **A**–**C** Flow cytometry analysis of germ-layer cell proportion changes following *TCF12*, *EBF1* and *NFIC* knockdown in EBs. **A** Experimental workflow for siRNA-mediated knockdown of *TCF12*, *EBF1* or *NFIC* in hESCs, followed by EB differentiation and flow cytometry analysis. **B** Representative flow cytometry histograms showing expression of *SOX17* & *GATA6* (endoderm), *EOMES* (mesoderm) and *PAX6* (ectoderm). **C** Paired dot plot showing SOX17^+^ & GATA6^+^ (endoderm), EOMES^+^ (mesoderm) and PAX6^+^ (ectoderm) cell percentages across groups (n ≥ 3 independent experiments; statistical significance was assessed using a paired t-test with * indicating *p*-value < 0.05). **D**–**F** Effects of *TCF12* knockdown in mesendoderm-directed differentiation culture system (MEM). **D** Schematic of germ-layer specification and knockdown efficiency after *TCF12* knockdown. **E** Immunofluorescence staining of control and *TCF12* knockdown cells (DAPI, blue). Scale bar: 25 µm. **F** RT-qPCR results showing the expression of lineage-specific TFs after knockdown. (G-I) Effects of *EBF1* knockdown in mesendoderm-directed differentiation culture system (MEM). **G** Schematic of germ-layer specification and knockdown efficiency after *EBF1* knockdown. **H** Immunofluorescence staining of control and *EBF1* knockdown cells (DAPI, blue). Scale bar: 25 µm. **I** RT-qPCR results showing the expression of lineage-specific TFs after knockdown. **J**–**L** Effects of *NFIC* knockdown in neural ectoderm-directed differentiation culture system (NIM). **J** Schematic of germ-layer specification and knockdown efficiency after *NFIC* knockdown. **K** Immunofluorescence staining of control and *NFIC* knockdown cells (DAPI, blue). Scale bar: 25 µm. **L** RT-qPCR results showing the expression of lineage-specific TFs after knockdown. NT indicates negative control. All RT-qPCR experiments were performed with 3 independent biological replicates. Data are presented as mean ± SEM. Statistical significance was assessed using an unpaired two-tailed Student’s t-test, which was defined as ns = not significant, *p < 0.05, **p < 0.01 and ***p < 0.001

Considering the network degree of TFs, their actual expression level, and chromatin accessibility in various experimental models, we individually selected three candidate TFs, which were HEB (TCF12), early B-cell factor 1 (EBF1) and nuclear factor I-C (NFIC). These three TFs were respectively associated with three intermediate stages. We then utilized small interfering RNA (siRNA) to disrupt the expression of these TFs in both spontaneous EB system and directed differentiation condition (Fig. 5).

Since spontaneous EBs consist of a mixed cell population, it is challenging to detect changes in specific subpopulations using RT-qPCR. Therefore, to assess the impact of knocking down germ-layer-associated TFs, we employed FACS to isolate cells belonging to the relevant germ layers based on established markers and quantified changes in the proportion of the target differentiated cell population (Fig. 5A). The results showed that knockdown of each of the three TFs led to a significant decrease in the proportion of cells in their respective germ layers, demonstrating that perturbation of the predicted TFs specifically altered the commitment levels of their corresponding germ-layer cells within the complex EB environment (Fig. 5B, C).

In addition, as each germ layer constitutes only a subset of the total cell population, making it difficult to distinguish the specific transcriptional impacts of perturbing a germ-layer-specific TF from background signals of other lineages, we further validated the effects of TF knockdown using the directed differentiation system, in which the majority of cells belong to the germ layer of interest. For the mesendoderm culture, we treated these cells with *TCF12* and *EBF1* siRNA for 48 h and collected RNA to confirm the efficiency (Fig. 5D–I). Interestingly, despite the morphological changes of cell population treated by siRNA targeting the distinct TFs, the endoderm/mesoderm markers noticeably decreased obviously, as revealed by the immunostaining and RT-qPCR (Fig. 5E, F, H, I). As Fig. 5F shows, *TCF12* knockdown group showed a dramatic fall in all endoderm-lineage specific markers (*SOX17*, *FOXA2*, *GATA6*) in the mRNA expression level, which was also validated by the immunostaining results (Fig. 5E), while there was a rise in the expression level of some mesoderm-lineage specific markers such as *T* and *MESP1*. At the same time, the expression level of mesodermal markers including *T*, *MESP1* and *MIXL1* were noticeably lower in the group treated with *EBF1* siRNA, and the expression level of *SOX17* as well as *GATA6* was significantly higher compared to the control group (Fig. 5I). In addition to the altered phenotype, the expression pattern of several target genes was also affected (Supplementary Fig. 7A-B). The cells treated with *TCF12* siRNA exhibited a preference to mesoderm differentiation, which is consistent with the previous observation in mouse that the depletion of *Tcf12* in mesendodermal cell led to differentiation towards mesodermal lineage [42]. Meanwhile, cell population after treatment with *EBF1* siRNA developed towards endodermal trajectory. In the case of neural differentiation, we treated cells with *NFIC* siRNA for 48 h and collected RNA to confirm the efficiency (Fig. 5J–L). Although the morphology of cell population treated by siRNA did not display a distinct change compared with that in control group, the ectoderm markers significantly decreased, as revealed by immunostaining and RT-qPCR (Fig. 5K-L). Moreover, we observed a changed expression pattern of several genes targeted by NFIC (Supplementary Fig. 7C).

In summary, the perturbation experiments confirmed the role of candidate TFs in early differentiation and validated the efficacy of establishing a TF regulatory network based on genomic information derived from scATAC-seq data.

## Discussion

In this study, we performed scATAC-seq on human ESC-derived EBs at two differentiation stages, identifying “early committed cells” of distinct germ layers from D4 to D8. This stage is crucial for gastrulation, where the bilaminar embryo transforms into a trilaminar structure–ectoderm, mesoderm, and endoderm–essential for organ formation. By establishing a transcriptional regulatory network that integrates TF properties and inferred loop signals, we revealed crucial TFs for early committed cells within each germ layer. Functional validation via siRNA knockdown confirmed their roles in fate determination, aligning with computational predictions.

Recent advancements in single-cell technology have enabled precise identification of cell states and transitions [43]. Using single-cell sequencing on early developmental models, we identified three populations of germ layer-specific early committed cells, with the transitional state population implicated in both development and pathology. Studying these unique cell populations helps decode developmental dynamics [44] and uncover pathogenetic mechanisms, inspiring potential therapeutic strategies targeting specific cellular components [45, 46]. However, identifying transitional cell populations has largely relied on single-cell transcriptome sequencing. In this study, we identified transitional cell populations with epigenetic characteristics from multiple cell types, based on TF binding motif information from scATAC-seq, without relying on expression pattern. Disrupting dominant TFs predicted to affect transitional populations resulted in characteristic phenotype, further validating our approach and confirming the efficiency and precision of our strategies.

Previous studies on human germ-layer differentiation in EB system have primarily focused on transcriptomic profiling. For example, Han et al. constructed a transcriptomic landscape of early differentiation lineages in EBs, revealing diverse cellular states across multiple cellular lineages [17]. Rhodes et al. further characterized the various cell types arising from EBs at later stages of differentiation, and demonstrated the utility of EBs as a useful model for investigating the regulatory events [47]. Although these scRNA-seq studies of EBs have revealed the heterogeneous cellular composition and developmental trajectories based on gene expression dynamics, investigations into chromatin accessibility changes during early human germ-layer differentiation remain limited. Notably, epigenetic changes can precede transcriptional changes and thus serve as earlier indicators of cell fate commitment [48]. To address this gap, our study complements prior findings by adopting the following approaches: (1) To explore the epigenetic mechanisms underlying gene regulation, we examined dynamic changes in chromatin accessibility using scATAC-seq. This approach offers complementary information on chromatin status and TF binding activity, as well as identifies accessible regulatory elements that correlate with and are responsible for spatiotemporal gene expression during lineage specification—thereby extending insights beyond conventional marker-based profiling. (2) To better interpret cell fate commitment from chromatin dynamics, we introduced a “consistency score” that integrates gene activity and TF binding variability to identify developmentally coherent cellular states. This framework thus provides an alternative basis for inferring early lineage commitment from epigenetic changes. (3) Beyond state identification, our in-depth analysis of accessible chromatin revealed higher-order genomic features, such as inferred enhancer loops and predicted TF target genes. This enabled us to construct a TF regulatory network based on a TF-loop-TF model, which helps elucidate potential regulatory circuits and prioritize candidate TFs for downstream functional studies. Therefore, by integrating these approaches, our work advances the understanding of early human germ-layer differentiation beyond transcriptional dynamics.

We observed early committed populations transitioning from pluripotency towards three germ layer specification, displaying chromatin characteristics of two cell types and revealing the continuous nature of germ layer-specific chromatin changes. Notably, our optimized scATAC-seq protocol, though yielding fewer cells, provided high-depth data for improved loop identification and motif analysis compared to high-throughput methods like 10 × Genomics. This enabled the prediction of cell-type-specific chromatin interaction. Furthermore, we identified novel functional TFs involved in lineage commitment by constructing a TF-TF regulatory network considering loop signals, which were validated in knockdown functional assays.

Here, we chose EB, three-dimensional aggregates representing the three primary germ layers, as a simplified model for investigating early germ layers specification. Various in vitro models, such as gastruloids [49] and organoids [50, 51], have been developed to study embryogenesis using the self-organization capacity of hESC. While these models offer valuable alternatives to human samples, their culture is time-consuming and challenging due to complex experimental conditions, including strict molecular supplement requirements. Previous studies using these models primarily focused on developmental dynamics, with few addressing the regulatory mechanisms of specific key event. In this study, we employed EBs cultured for nearly a week in E6 medium under suspension culture to model early germ layer specification. Using this model, we identified several important TFs, such as TCF12, EBF1 and NFIC, involved in early lineage commitment. Notably, these TFs have also been reported to play roles in lineage commitment. For example, Li et al. reported that TCF12 deletion disrupted mesodermal development and reduced mesendodermal fate regulator expression during hematopoietic differentiation [52]. EBF1 is essential for B-cell differentiation [53] and directs mesenchymal stem cells toward adipocytes [54, 55] and osteoblasts lineages [56], highlighting its importance in mesodermal lineage commitment. NFIC has been linked to neurodevelopmental disruptions. Huang et al. observed that NFIC upregulation activated miR-200b, causing neural tube defects in mice [57]. Additionally, Brun et al. found that the NFI family, including NFIC, negatively regulated HEY1, which is associated with glioblastoma properties [58]. Unlike these studies focusing on later developmental stages, our work using EBs highlights the critical roles of these TF in early germ layer specification.

Some conclusions of our study were constrained by technical limitations. The plate-based scATAC-seq system used here yields low cell numbers and lacks expression data. Future studies should enhance throughput using combinatorial indexing [20], and integrate with expression profiling. Furthermore, although clustering strategy based on consistency scores effectively captures cell fate differentiation, further improvements on clustering performance could be explored, including optimizing the relative weighting between gene activity and binding activity, as well as improving the robustness of the consistency score. Additionally, our functional experiments relied on siRNA knockdown in cells under directed differentiation culture system rather than CRISPR/Cas9-mediated gene knockout, which limits long-term observations. High throughput screening in the EB model followed by in vivo validation is recommended. Integrating single-cell sequencing with perturbation experiments could further clarify genotype–phenotype relationships [59, 60]. Improvements in loop inference and multi-omics analysis are also needed to refine TF-TF interaction predictions. In conclusion, our scATAC-seq analysis reveals dynamic chromatin changes and novel TFs critical for early developmental stages.

## Conclusions

Our findings reveal that scATAC-seq can proficiently identify transitional cell populations and key TFs critical for early germ layer determination. Additionally, our methodological framework provides a valuable guide for future research in this complex field, facilitating mechanistic exploration. Overall, this work enhances our understanding of cell differentiation and germ layer specification, potentially advancing developmental biology research.

## Supplementary Information

Supplementary file 1. Quality assessment of Plate-based scATAC-seq library.(A) The peak signal identified in Day 4 and Day 8 EBs at a locus containing the TSS of germ layer-specific markers. y-axis: Norm. ATAC signal range. (B) The distribution of peaks in the genome. (C) Fragment counts (left) and TSS reads (right) versus TSS enrichment score.

Supplementary file 2. Joint assessment of the consistency between gene activity and binding activity of germ layer specific TFs.(A-C) Heatmaps showing the gene activity (A), binding activity (B) and consistency score (C) of germ layer specific TFs in each cell.

Supplementary file 3. Identification of novel TFs in the ESC, END, MES and ECT.(A-C) Stacked violin plot showing the consistency score of the top 10 TFs that are specifically higher in END (A), MES (B) and ECT (C), respectively. (D) The TF-TF regulatory network in END (Left), MES (Middle) and ECT (Right).

Supplementary file 4. UMAP and LDA visualization of key cell-type-specific TFs.(A) Labelling of cell types on UMAP (left) and LDA plot (right). (B) Visualization of consistency scores of key cell-type-specific TFs on UMAP and LDA plot.

Supplementary file 5. Visualization of motif enrichment, ATAC peak accessibility and InferLoop-predicted loops at loci of germ-layer-specific genes.Genome browser view showing scATAC-seq chromatin accessibility signals (EB Day 4), enriched motif positions (red vertical ticks), and inferred chromatin loops at three representative loci: FOXA2 (TCF12 motifs), MESP1 (EBF1 motifs), and PAX6 (NFIC motifs). Blue arcs indicate all chromatin loops predicted by InferLoop; red arcs highlight loops whose associated peak positions overlap with the respective TF binding motifs (TCF12, EBF1, or NFIC). Sequence logos (bits scale) and DNA sequences at overlapped positions are displayed below each browser panel. Scale bars: 5 kb.

Supplementary file 6. Establishment of the directed differentiation system of hESC.(A-C) The culture system directing hESC differentiation into mesendoderm cells (A), validated by RT-qPCR (B) and immunostaining (C) of specific markers. (B) RT-qPCR results showing the expression of mesendoderm-specific markers. (C) Immunostaining of mesendoderm-specific markers. Nuclei were counterstained with DAPI (blue). Scale bar = 25 μm. (D-F) The culture system directing hESC differentiation into neural ectoderm cells (D),39validated by RT-qPCR (E) and immunostaining (F) of specific markers. (E) RT-qPCR results showing the expression of neural ectoderm-specific markers. (C) Immunostaining of neural ectoderm-specific markers. Nuclei were counterstained with DAPI (blue). Scale bar = 25 μm. All RT-qPCR experiments were performed with 3 independent biological replicates. Data are presented as mean ± SEM. Statistical significance was assessed using an unpaired two-tailed Student's t-test, which was defined as *p < 0.05, **p < 0.01 and ***p < 0.001.

Supplementary file 7. Expression analysis of the predicated target genes upon the knockdown of TCF12, EBF1 and NFIC in respective culture systems.The expression level of target genes is assessed by RT-qPCR after perturbation experiments involving the knockdown of TCF12 (si-TCF12) (A) and EBF1 (si-EBF1) (B) in mesoderm differentiation system, and the knockdown of NFIC (si-NFIC) in neural ectoderm differentiation system (C). si-NT indicates negative control. All data are presented as mean ± SEM from n = 3 independent experiments. Statistical significance was determined by an unpaired two-tailed Student's t-test compared with si-NT group, which was defined as *p < 0.05, **p < 0.01, ***p < 0.001 and ****p < 0.0001.

Supplementary Table S1

Supplementary Table S2

Supplementary Table S3

## Data Availability

The codes used for analyses in this study are deposited on Github (https://github.com/Li-Lab2shsmu/scATAC-seq_analysis_of_human_EBs) under the GPL-3.0 license. The published zebrafish multi-omics dataset can be accessed through the accession number GSE223636 [61], with the zebrafish motif annotation collected the DANIO-CODE database [62, 63]. The sequencing data supporting the results presented in this study are deposited in China National Center for Bioinformation (https://www.cncb.ac.cn/) (GSA-human: HRA010608).

## Abbreviations

TF: Transcription factor
InferLoop: Inferred loop signals
hESC: Human embryonic stem cell
ICM: Inner cell mass
EB: Embryoid body
ME: Mesendoderm
siRNA: Small interfering RNA
NT: Negative control
FACS: Fluorescence activated cell sorting
cDNA: Complementary DNA
qPCR: Quantitative PCR
hPSCs: Human pluripotent stem cells
SDS: Sodium dodecyl sulfate
TSS: Transcription start site
UMAP: Uniform Manifold Approximation and Projection
TG: Target gene
RREB1: Ras-responsive element binding protein 1
ZNF460: Zinc finger protein 460
KLF7: Krüppel-like factor 7
NFIC: Nuclear Factor I C
TCF12: Transcription Factor 12
EBF1: EBF Transcription Factor 1, Early B-cell factor 1
NANOG: Nanog homeobox
SOX17: SRY-Box Transcription Factor 17
POU5F1: POU class 5 homeobox 1
FOXA2: Forkhead Box A2
MIXL1: Mix Paired-Like Homeobox
MESP1: Mesoderm Posterior bHLH Transcription Factor 1
PAX6: Paired box gene 6
SOX1: SRY-Box Transcription Factor 1
TFAP2A: Transcription Factor AP-2 Alpha
GRHL2: Grainyhead Like Transcription Factor 2
DE: Definitive endoderm
IF: Immunofluorescence
CS: Carnegie Stages
NTDs: Neural tube defects
GBMs: Glioblastomas
