# Supplementary figures and images for: Single-cell ATAC-seq analysis of human embryoid bodies reveals crucial transcription factor networks involved in early germ layer specification
Source: Cell Biosci. 2026 Mar 29;16:54. doi: 10.1186/s13578-026-01561-8 (PMC13154528; doi:10.1186/s13578-026-01561-8)

**A**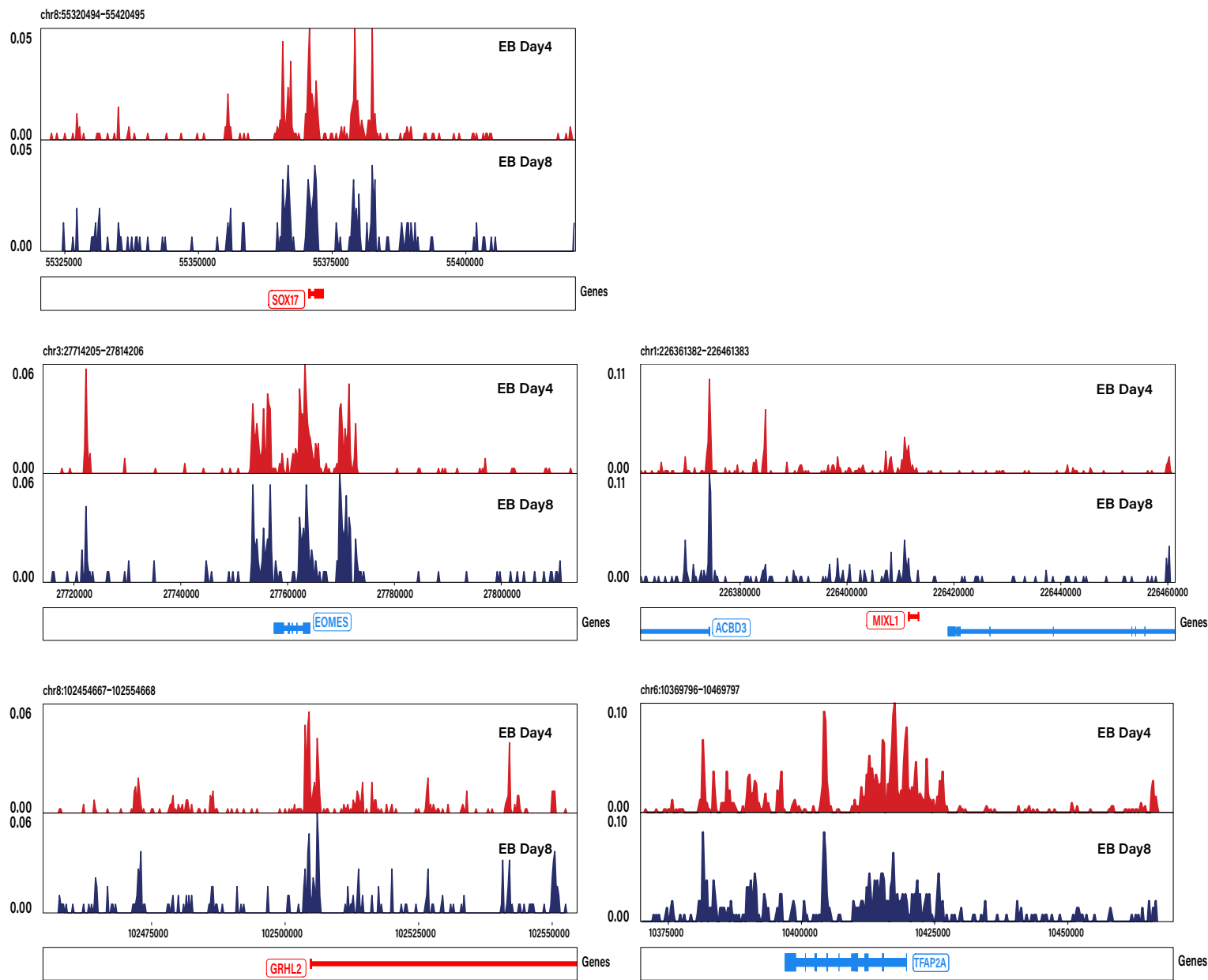**B**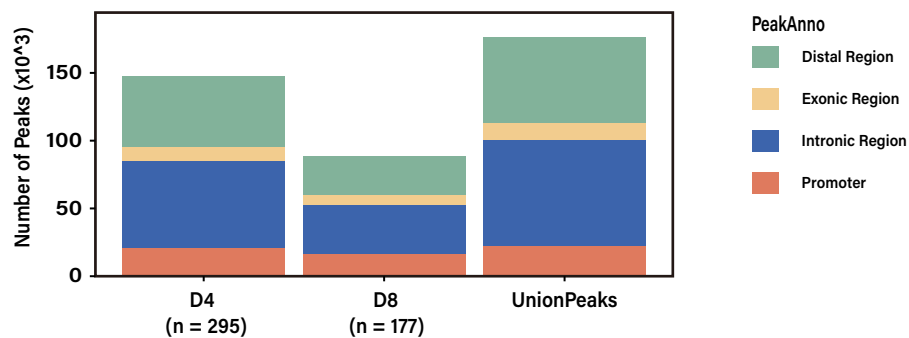**C**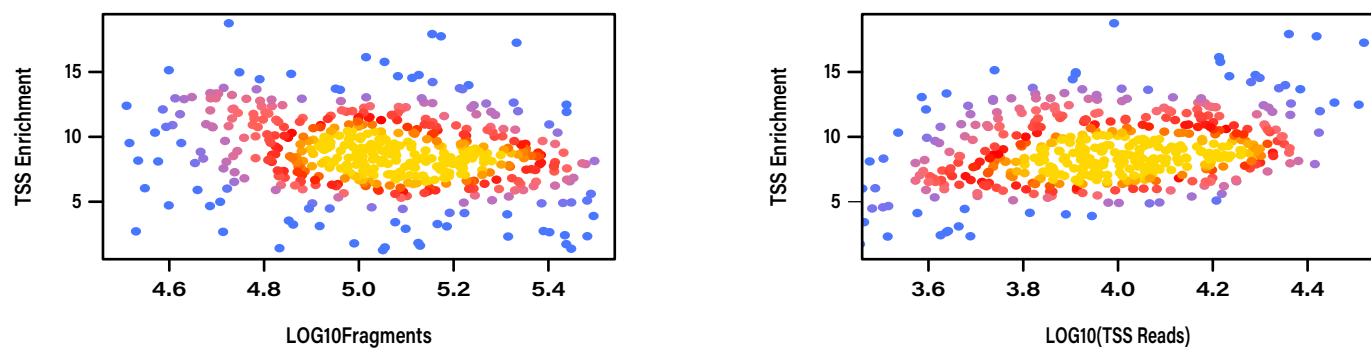

Supplement: Supplementary file 1 — Quality assessment of Plate-based scATAC-seq library.(A) The peak signal identified in Day 4 and Day 8 EBs at a locus containing the TSS of germ layer-specific markers. y-axis: Norm. ATAC signal range. (B) The distribution of peaks in the genome. (C) Fragment counts (left) and TSS reads (right) versus TSS enrichment score. [file 13578_2026_1561_MOESM1_ESM.pdf]

A

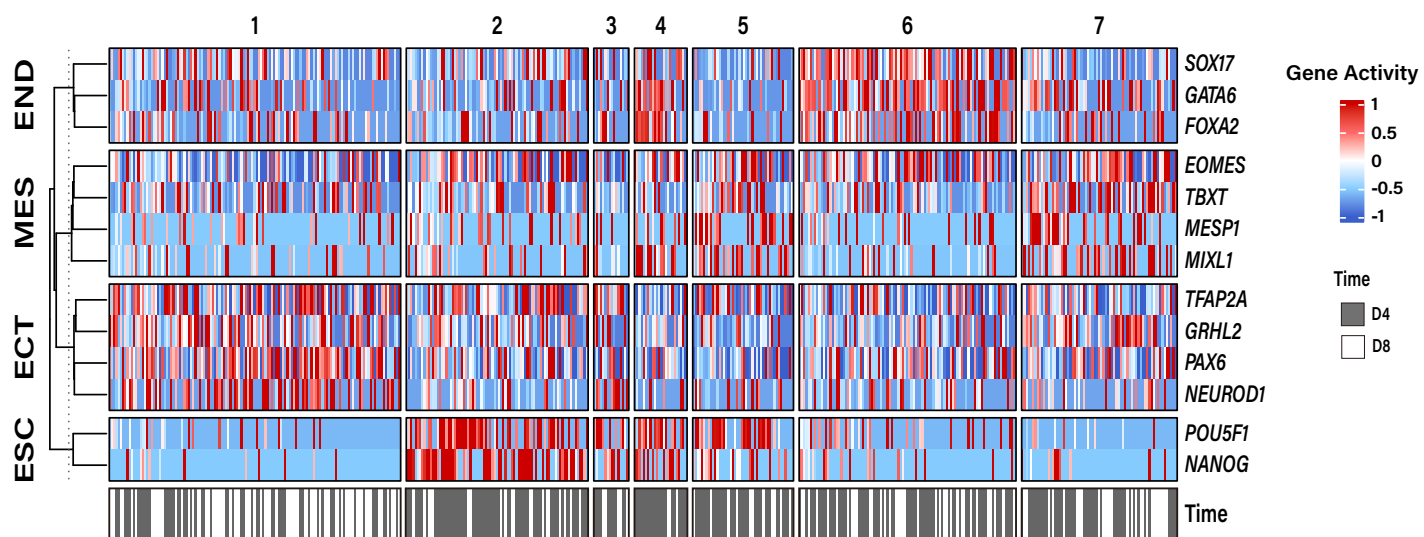

B

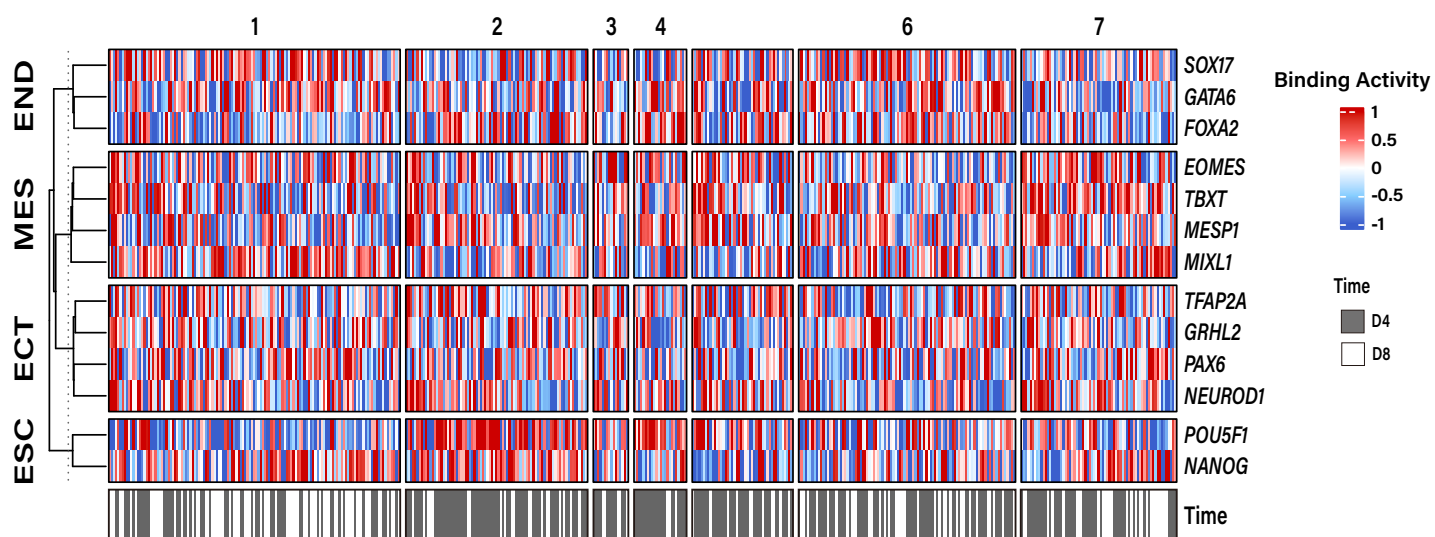

C

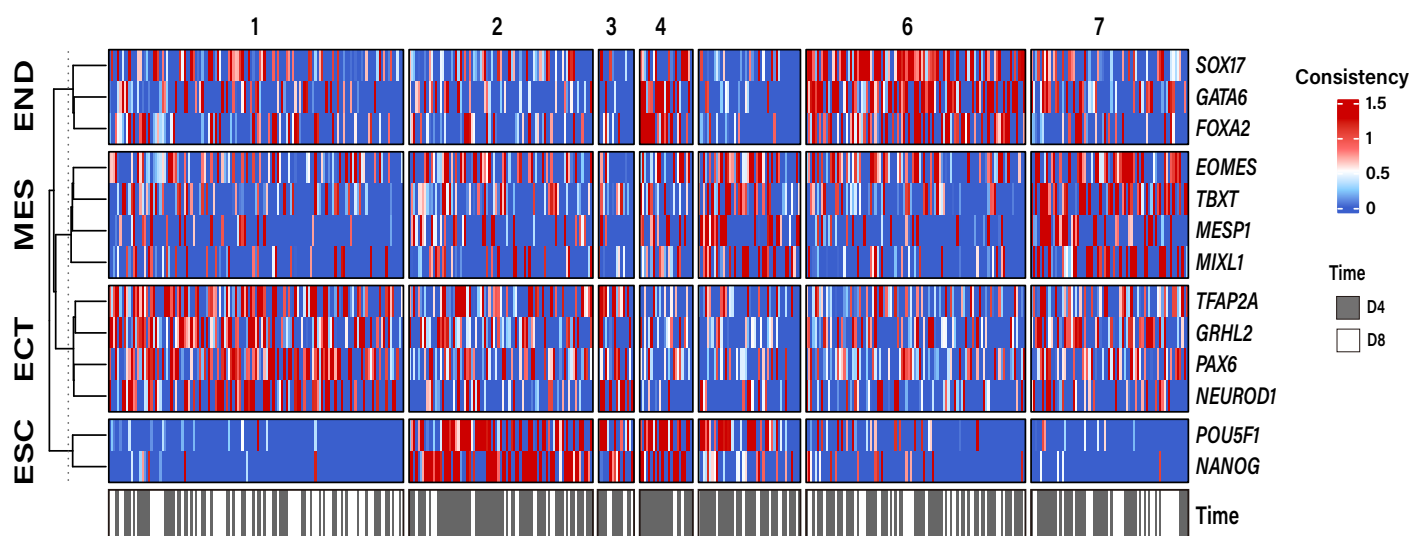

Supplement: Supplementary file 2 — Joint assessment of the consistency between gene activity and binding activity of germ layer specific TFs.(A-C) Heatmaps showing the gene activity (A), binding activity (B) and consistency score (C) of germ layer specific TFs in each cell. [file 13578_2026_1561_MOESM2_ESM.pdf]

**A**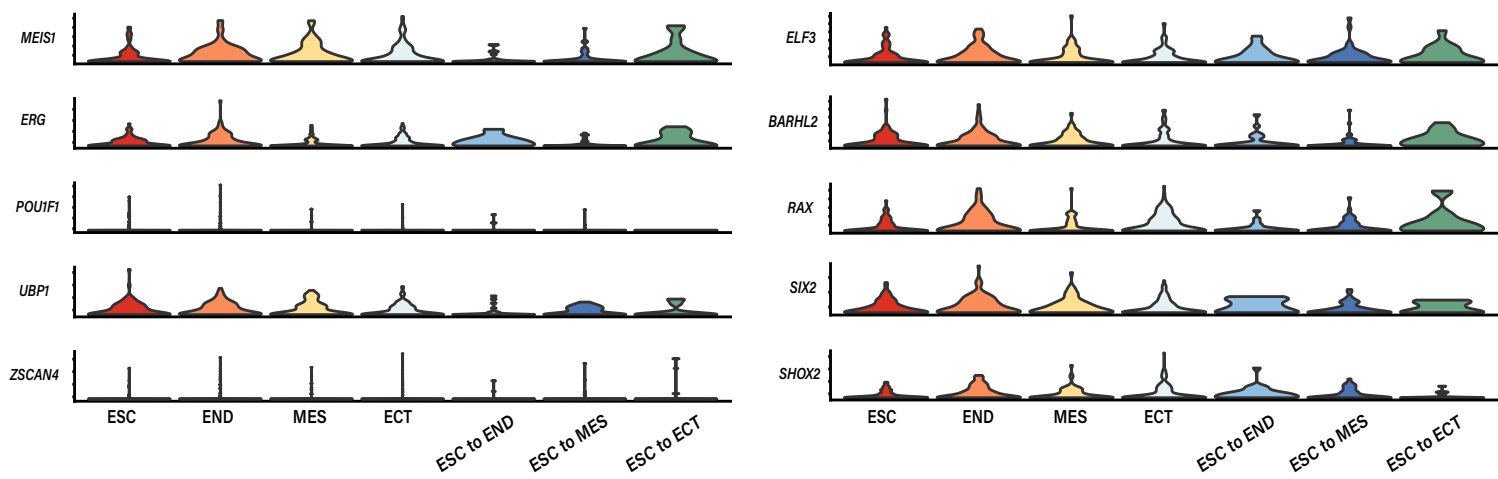**B**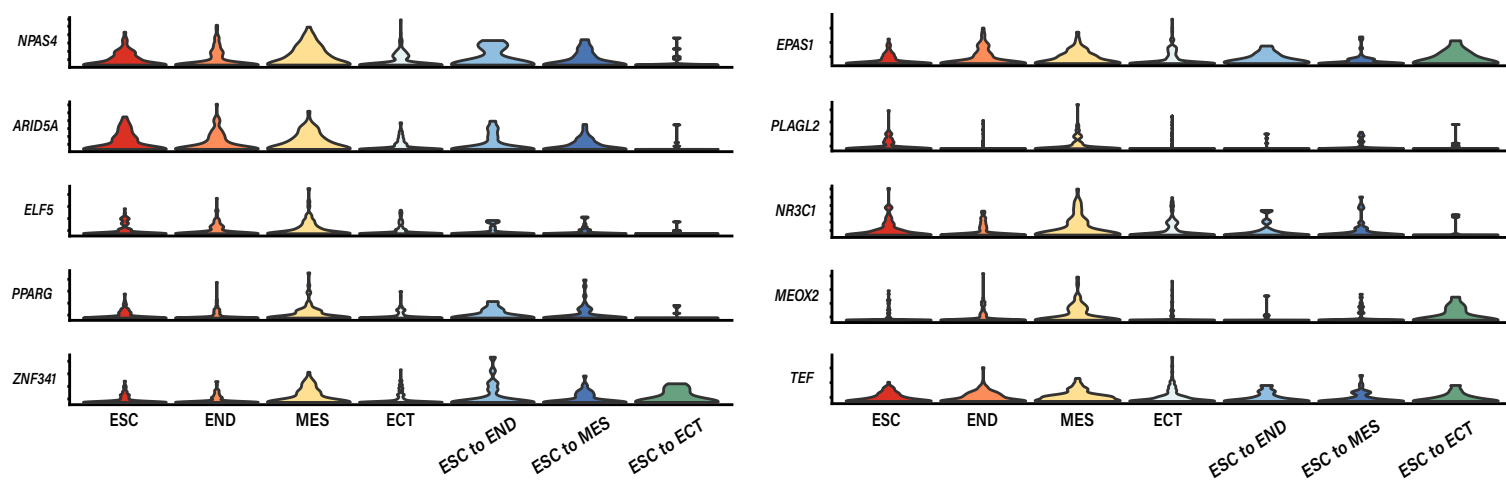**C**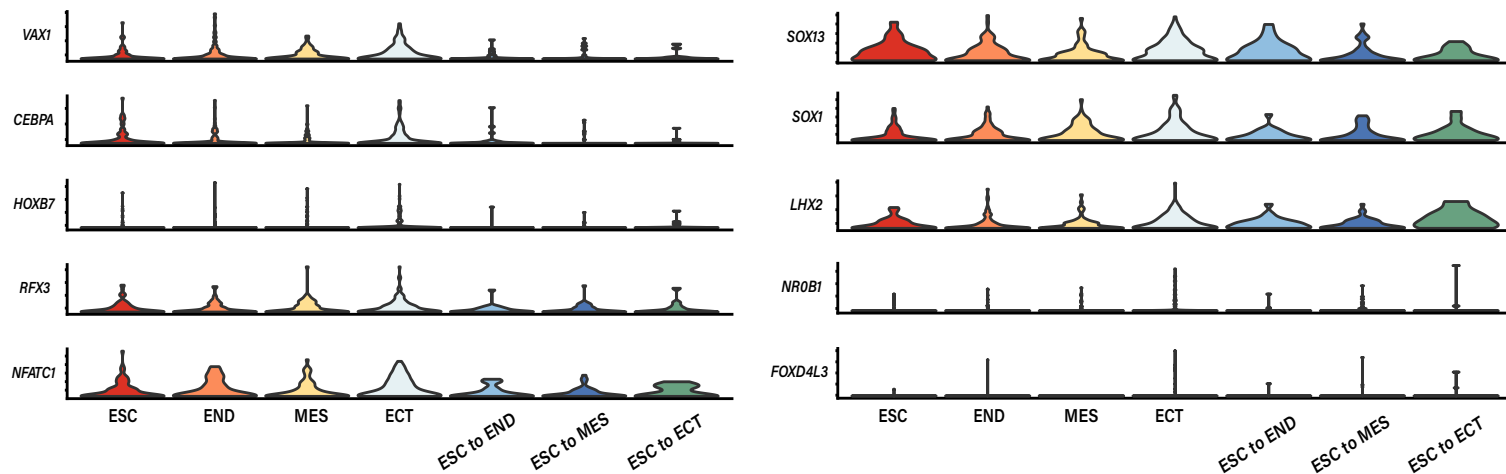**D**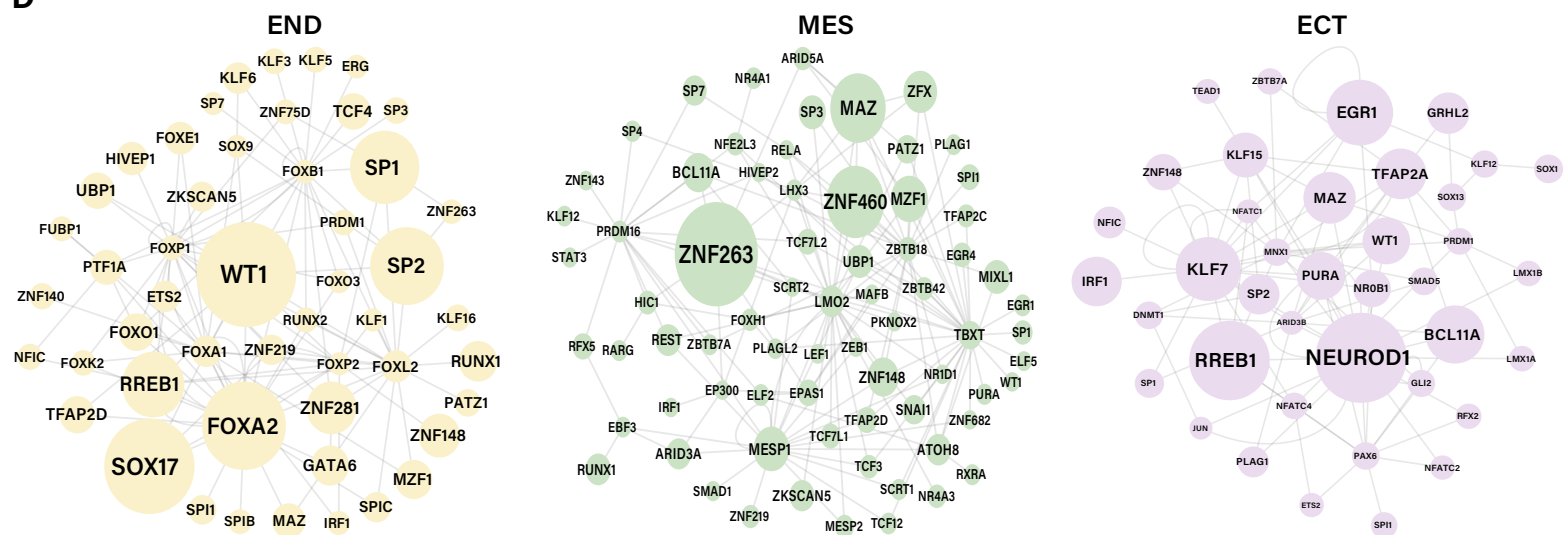

Supplement: Supplementary file 3 — Identification of novel TFs in the ESC, END, MES and ECT.(A-C) Stacked violin plot showing the consistency score of the top 10 TFs that are specifically higher in END (A), MES (B) and ECT (C), respectively. (D) The TF-TF regulatory network in END (Left), MES (Middle) and ECT (Right). [file 13578_2026_1561_MOESM3_ESM.pdf]

**A**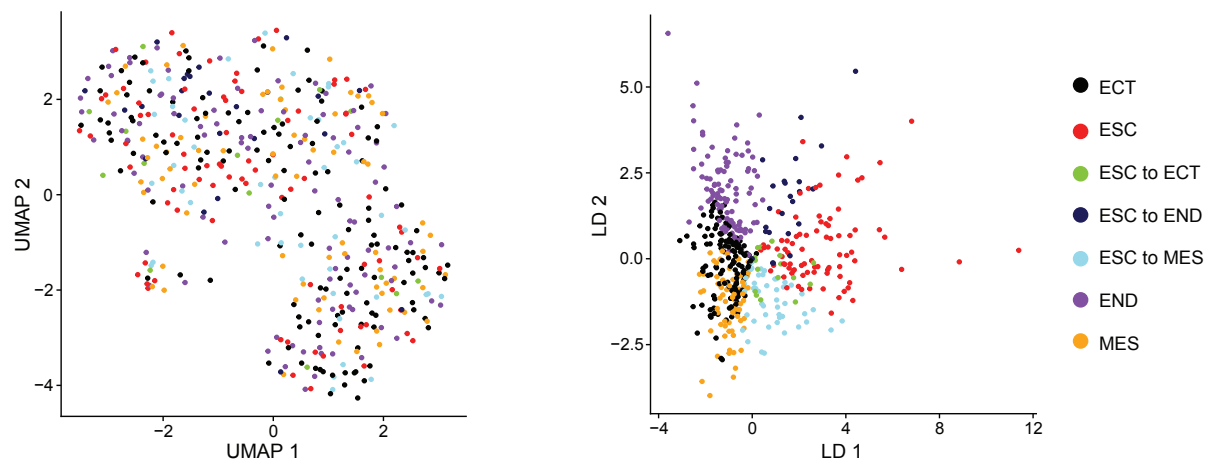**B**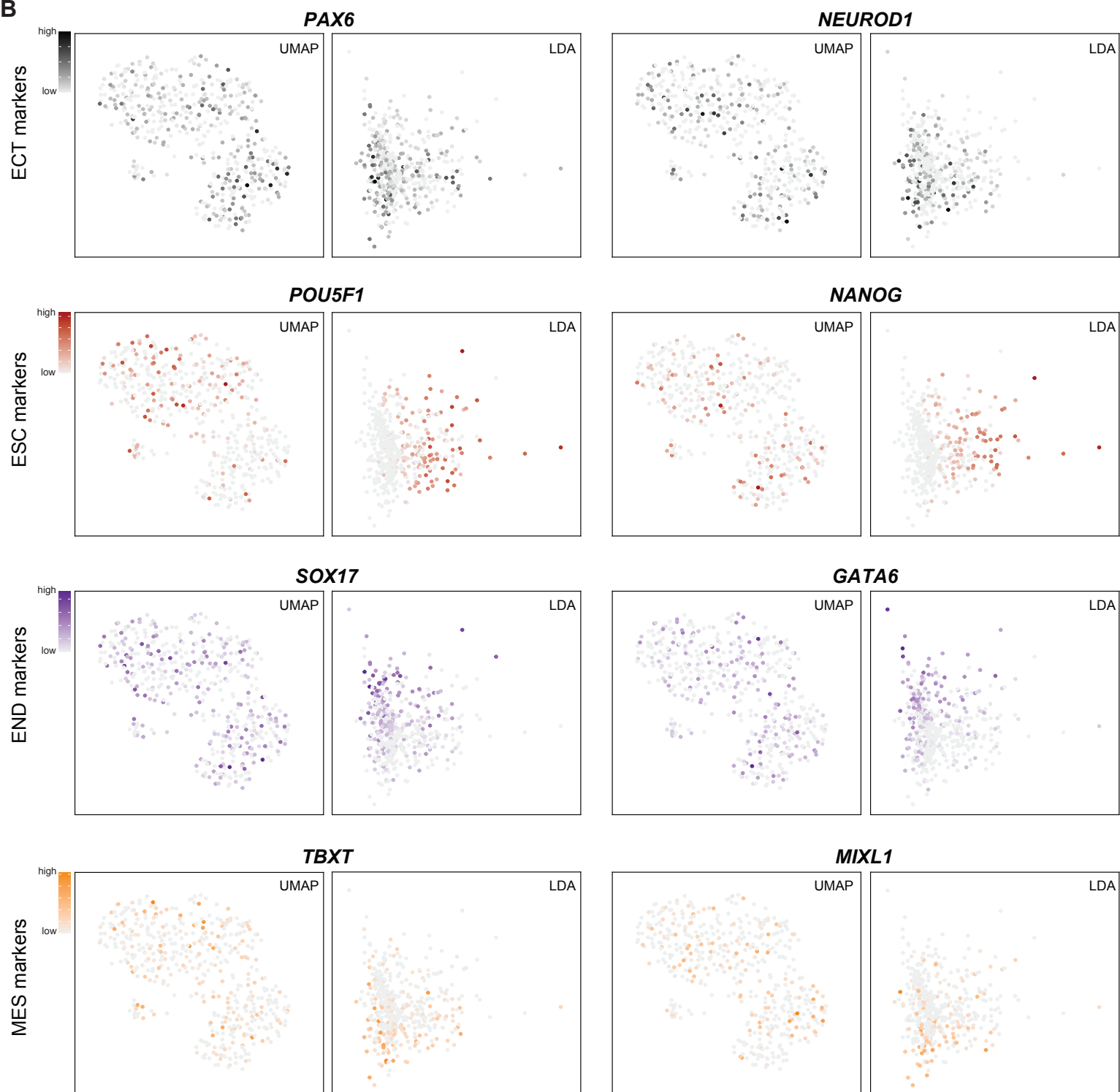

Supplement: Supplementary file 4 — UMAP and LDA visualization of key cell-type-specific TFs.(A) Labelling of cell types on UMAP (left) and LDA plot (right). (B) Visualization of consistency scores of key cell-type-specific TFs on UMAP and LDA plot. [file 13578_2026_1561_MOESM4_ESM.pdf]

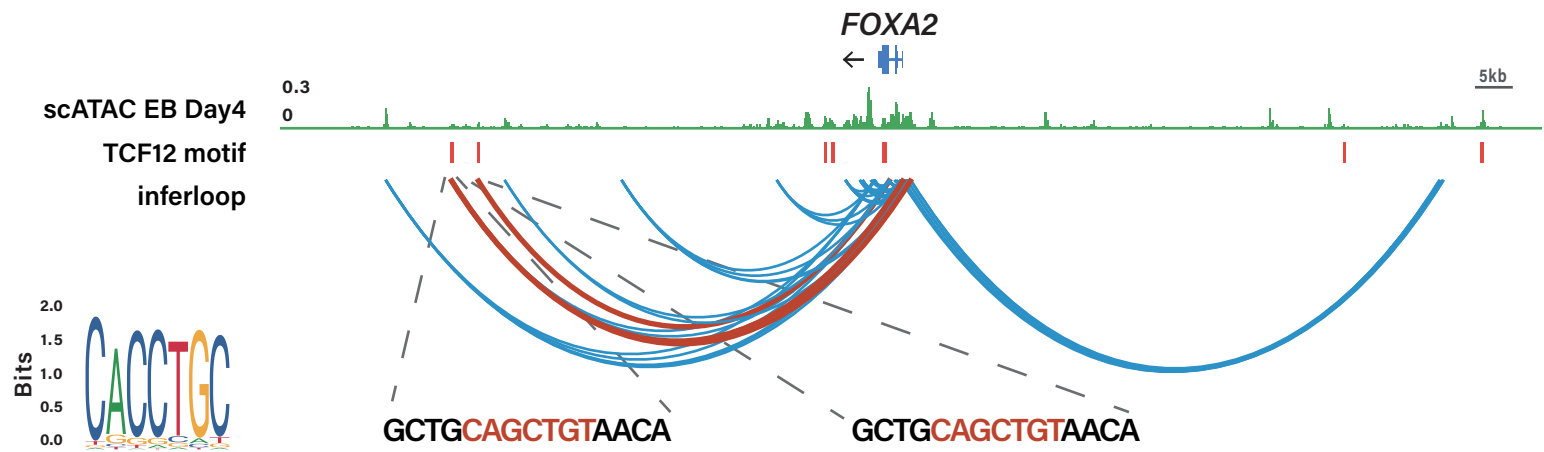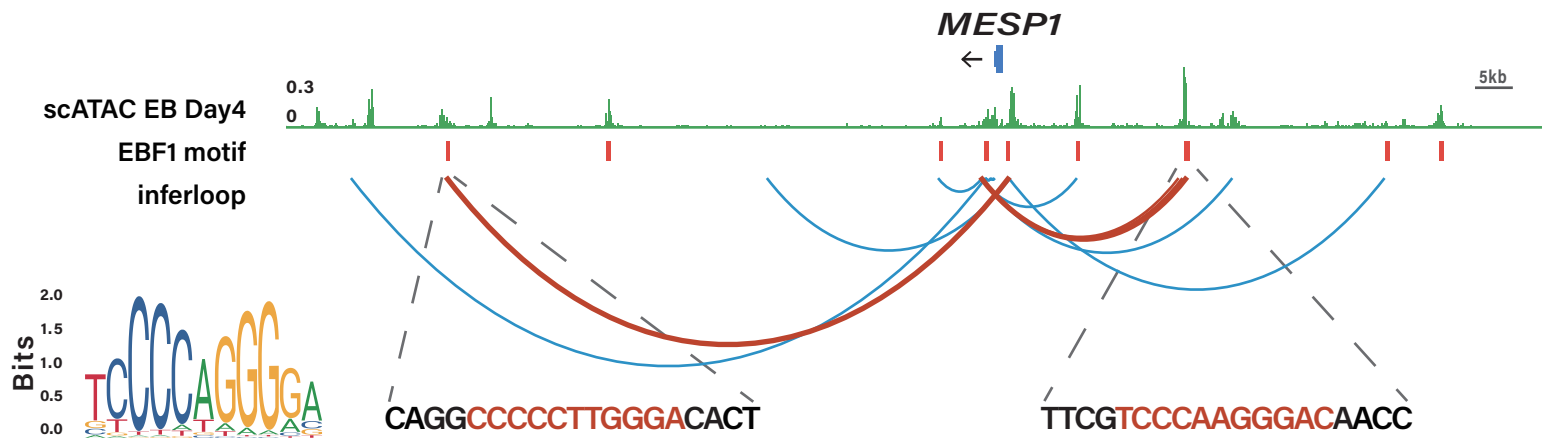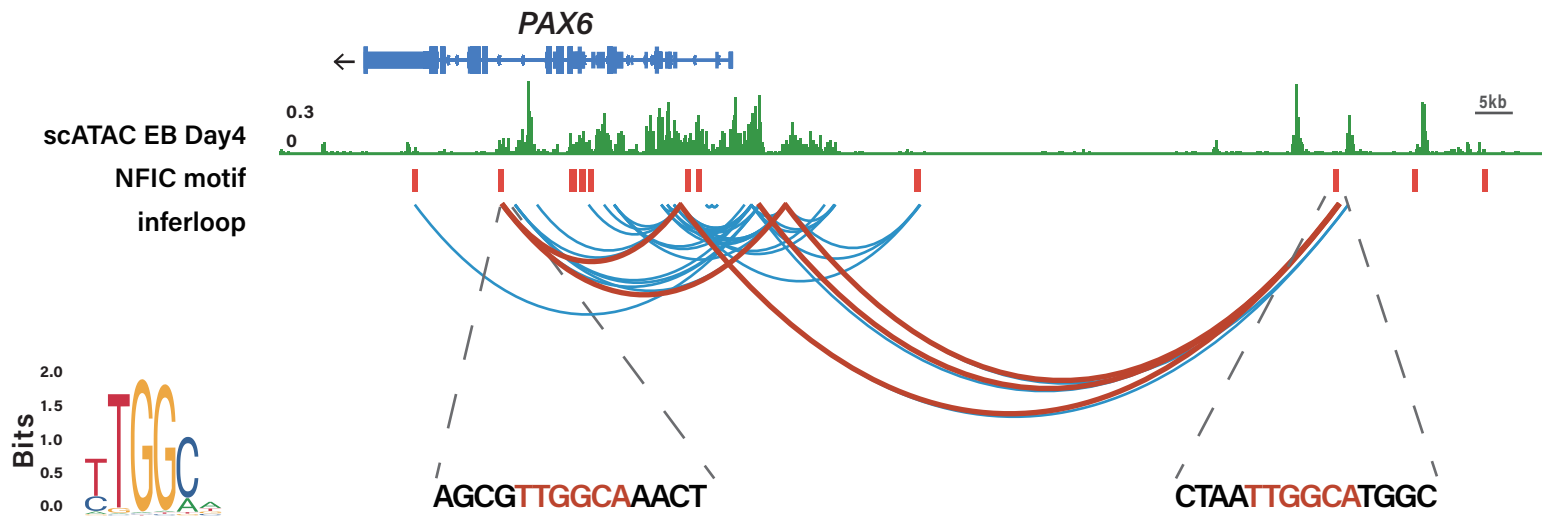

Chromatin loops predicted by inferLoop

Predicted loops overlapping with TCF12/EBF1/NFIC motifs

Supplement: Supplementary file 5 — Visualization of motif enrichment, ATAC peak accessibility and InferLoop-predicted loops at loci of germ-layer-specific genes.Genome browser view showing scATAC-seq chromatin accessibility signals (EB Day 4), enriched motif positions (red vertical ticks), and inferred chromatin loops at three representative loci: FOXA2 (TCF12 motifs), MESP1 (EBF1 motifs), and PAX6 (NFIC motifs). Blue arcs indicate all chromatin loops predicted by InferLoop; red arcs highlight loops whose associated peak positions overlap with the respective TF binding motifs (TCF12, EBF1, or NFIC). Sequence logos (bits scale) and DNA sequences at overlapped positions are displayed below each browser panel. Scale bars: 5 kb. [file 13578_2026_1561_MOESM5_ESM.pdf]

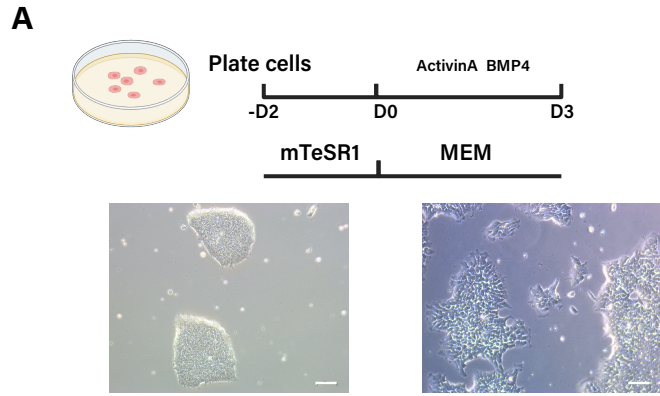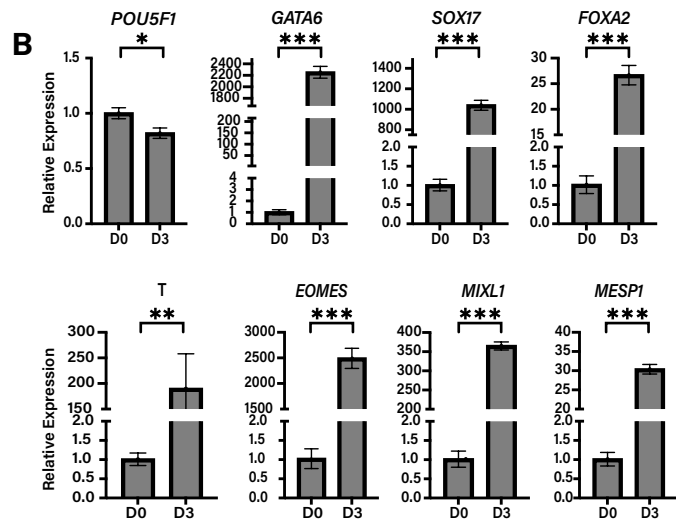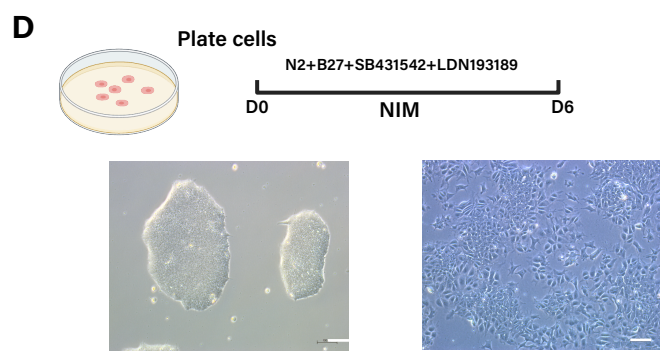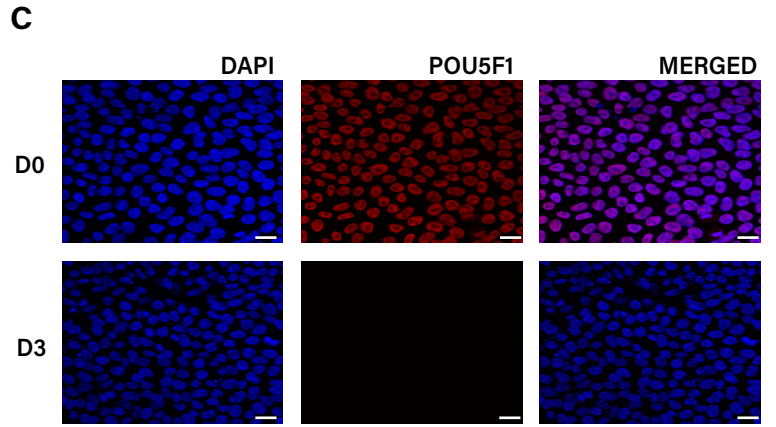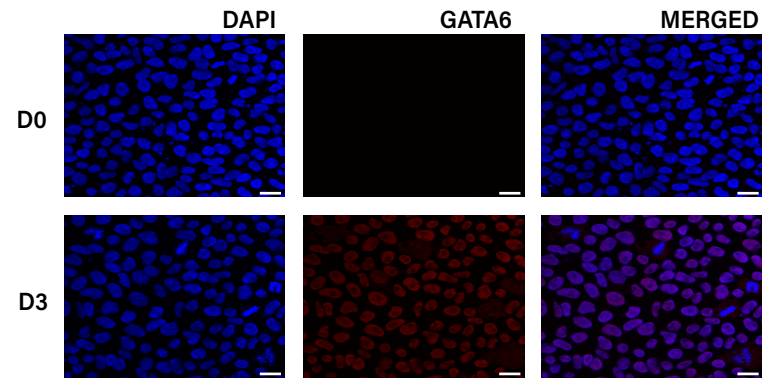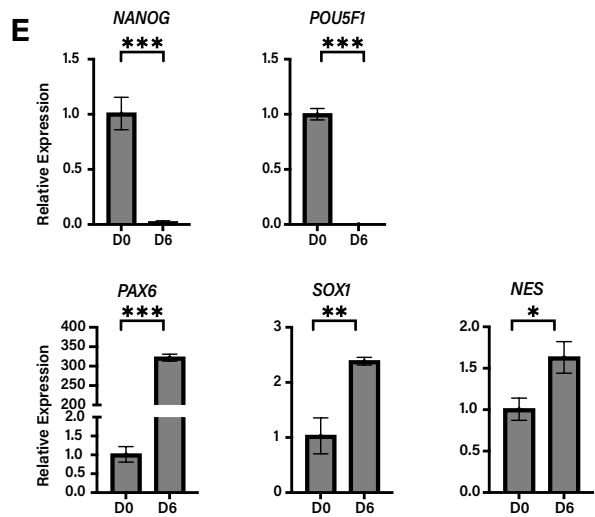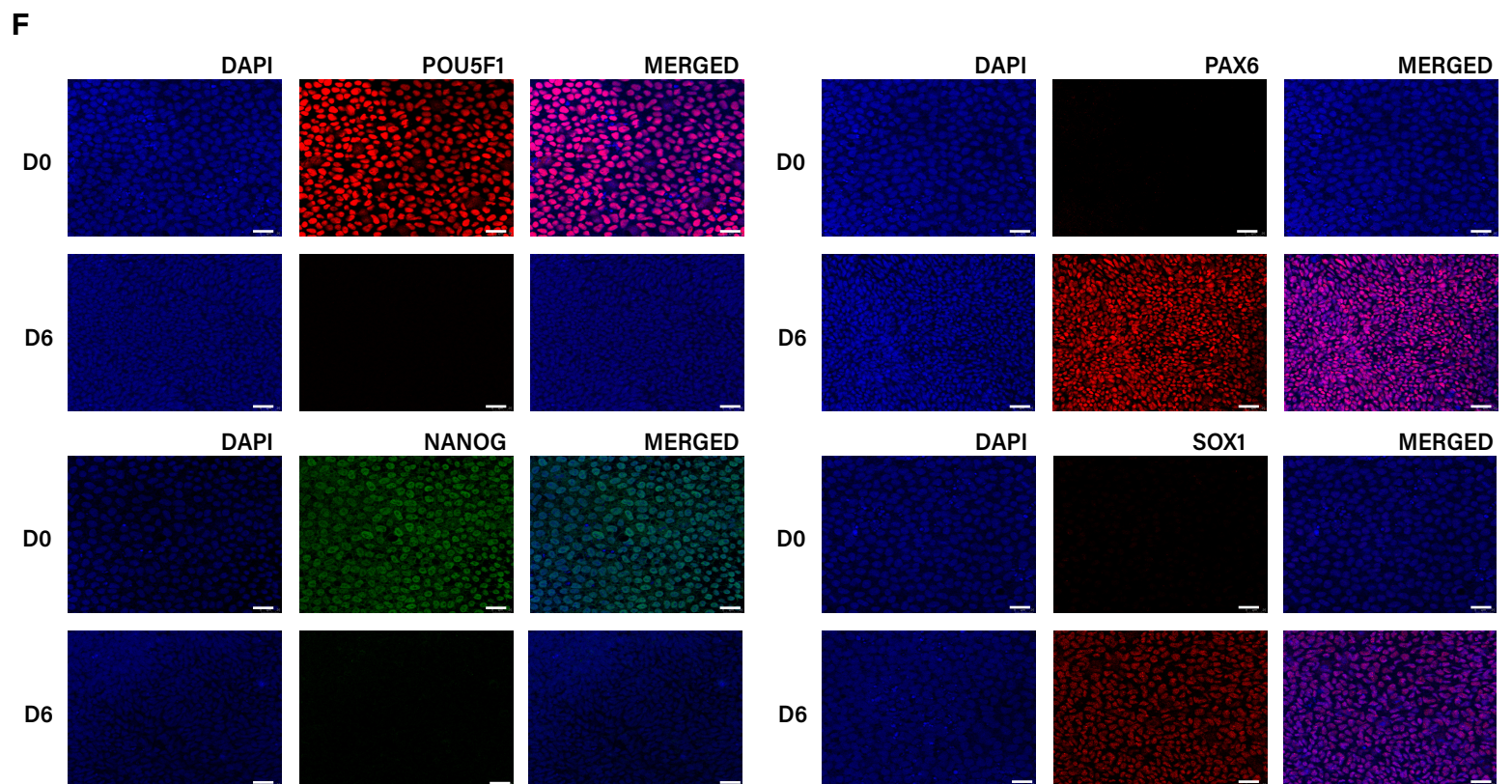

Supplement: Supplementary file 6 — Establishment of the directed differentiation system of hESC.(A-C) The culture system directing hESC differentiation into mesendoderm cells (A), validated by RT-qPCR (B) and immunostaining (C) of specific markers. (B) RT-qPCR results showing the expression of mesendoderm-specific markers. (C) Immunostaining of mesendoderm-specific markers. Nuclei were counterstained with DAPI (blue). Scale bar = 25 μm. (D-F) The culture system directing hESC differentiation into neural ectoderm cells (D),39validated by RT-qPCR (E) and immunostaining (F) of specific markers. (E) RT-qPCR results showing the expression of neural ectoderm-specific markers. (C) Immunostaining of neural ectoderm-specific markers. Nuclei were counterstained with DAPI (blue). Scale bar = 25 μm. All RT-qPCR experiments were performed with 3 independent biological replicates. Data are presented as mean ± SEM. Statistical significance was assessed using an unpaired two-tailed Student's t-test, which was defined as *p < 0.05, **p < 0.01 and ***p < 0.001. [file 13578_2026_1561_MOESM6_ESM.pdf]

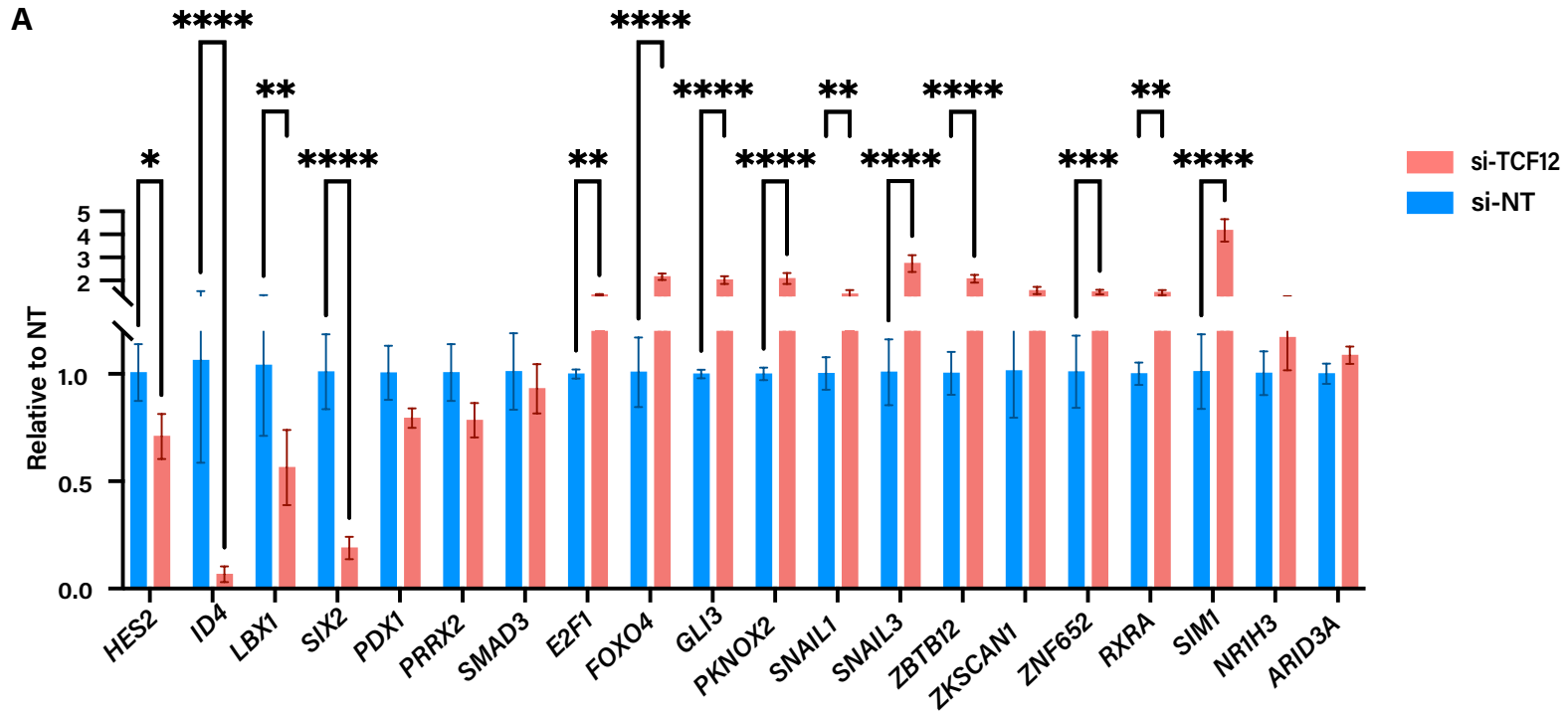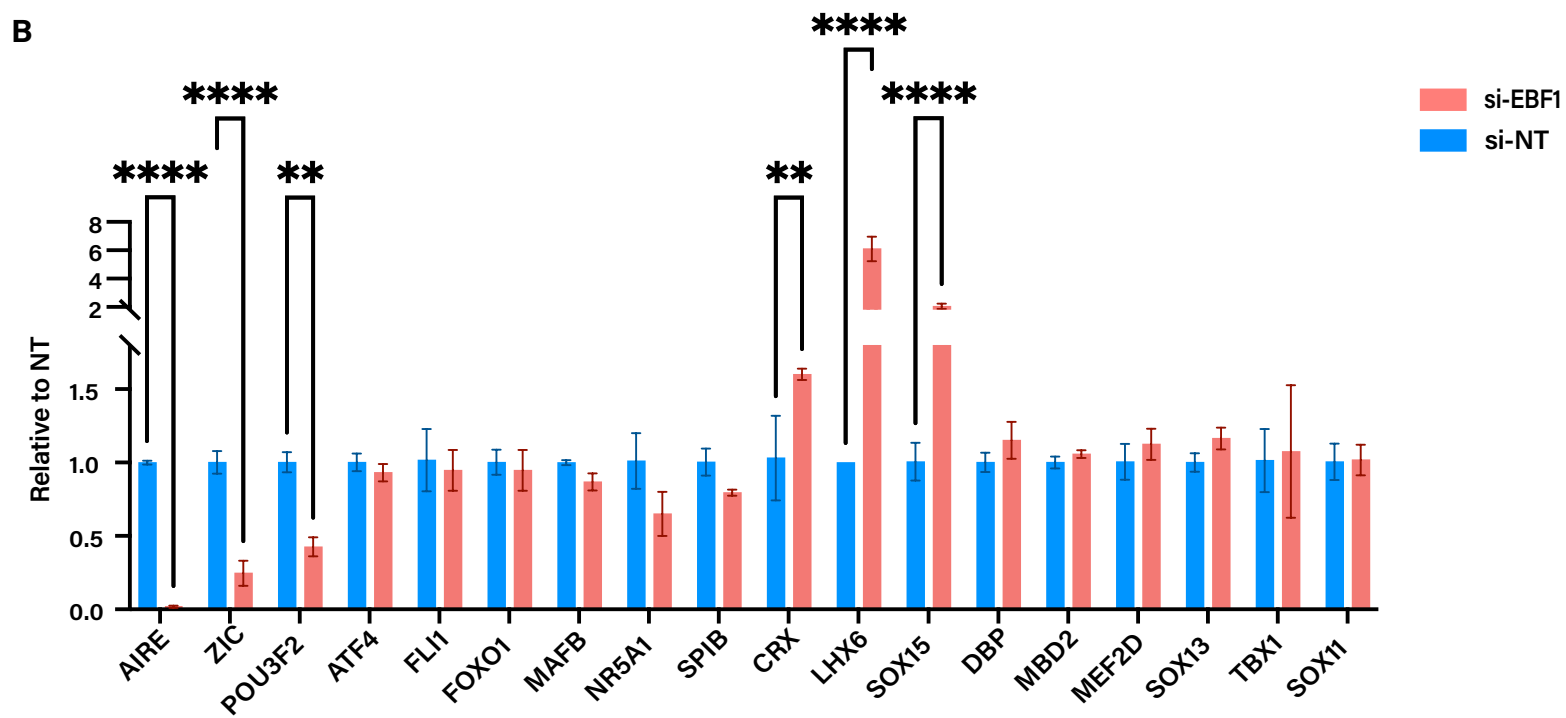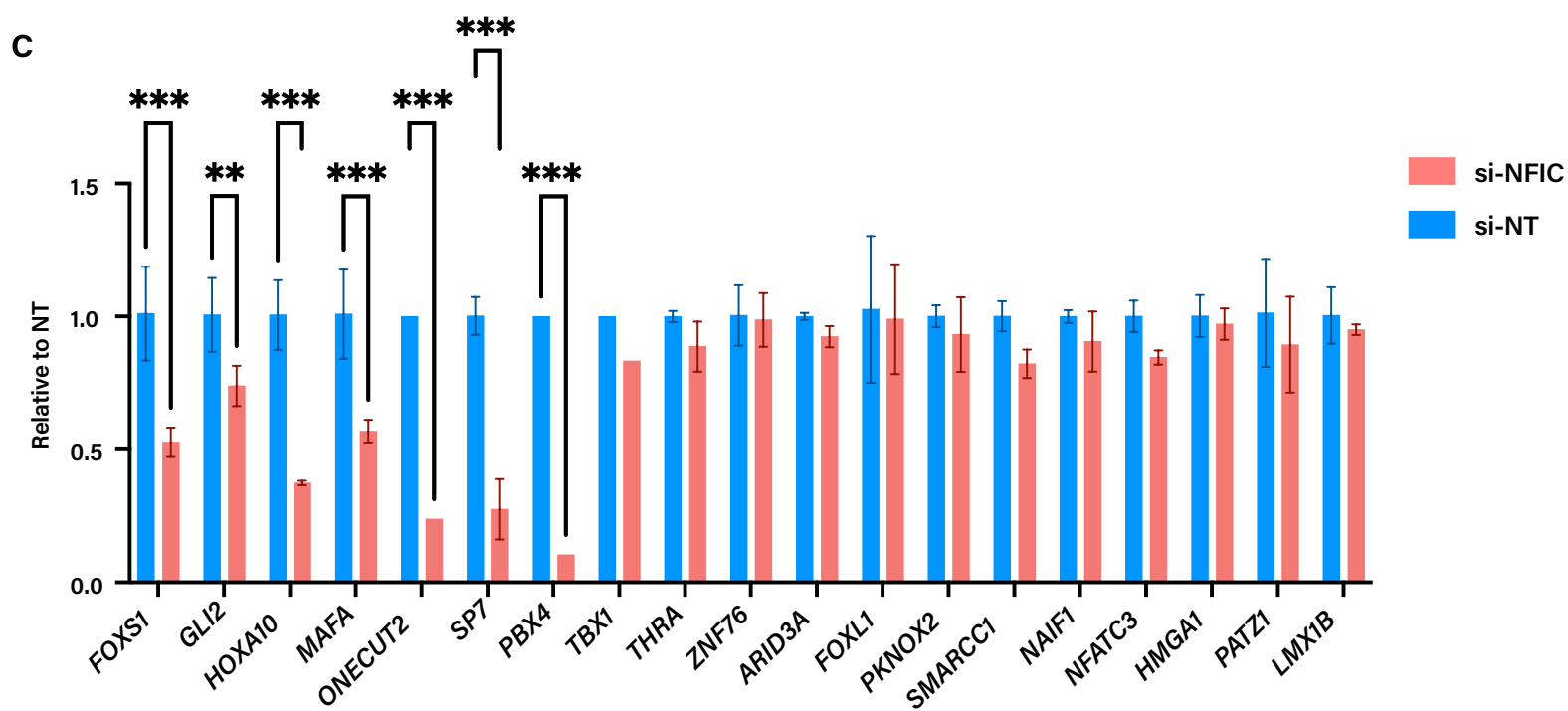

Supplement: Supplementary file 7 — Expression analysis of the predicated target genes upon the knockdown of TCF12, EBF1 and NFIC in respective culture systems.The expression level of target genes is assessed by RT-qPCR after perturbation experiments involving the knockdown of TCF12 (si-TCF12) (A) and EBF1 (si-EBF1) (B) in mesoderm differentiation system, and the knockdown of NFIC (si-NFIC) in neural ectoderm differentiation system (C). si-NT indicates negative control. All data are presented as mean ± SEM from n = 3 independent experiments. Statistical significance was determined by an unpaired two-tailed Student's t-test compared with si-NT group, which was defined as *p < 0.05, **p < 0.01, ***p < 0.001 and ****p < 0.0001. [file 13578_2026_1561_MOESM7_ESM.pdf]
